# Kinetic-driven microphase separation and vitrification of crowded hydrogels with associative bonds

**DOI:** 10.1126/sciadv.aed8808

**Published:** 2026-10-16

**Authors:** Dong Shi, Ning Li, Koichi Mayumi, Xiang Li, Takayuki Nonoyama, Jian Ping Gong

**Affiliations:** ^1^ Faculty of Advanced Life Science, Hokkaido University, Kita 21 Nishi 11, Kita-ku, Sapporo 001-0021, Japan.; ^2^ Graduate School of Life Science, Hokkaido University, Kita 21 Nishi 11, Kita-ku, Sapporo 001-0021, Japan.; ^3^ The Institute for Solid Physics (ISSP), The University of Tokyo, 5-1-5 Kashiwanoha, Kashiwa, Chiba 277-8581, Japan.; ^4^ Co-Creation Core for Soft Materials Aspiring Research & Translation (C^3^-SMART), Hokkaido University, Kita 21 Nishi 11, Kita-ku, Sapporo 001-0021, Japan.; ^5^ Institute for Chemical Reaction Design and Discovery (WPI-ICReDD), Hokkaido University, Kita 21 Nishi 11, Kita-ku, Sapporo 001-0021, Japan.

## Abstract

The thermal response of stimuli-responsive hydrogels is typically viewed through thermodynamic equilibrium. However, in “crowded” networks with dense associative bonds, elasticity and diffusion kinetics dictate distinct nonequilibrium fates. We investigate thermal transitions of a PAAc-CaAc_2_ (polyacrylic acid complexed with calcium ions) hydrogel along two trajectories: a quasi-static isobaric pathway (path A) and a rapid quasi-isochoric pathway (path B). Along path A, the gel contracts homogeneously until reaching a hardening temperature via global vitrification (*T*_hard_). In contrast, path B triggers confined microphase separation into polymer-dense and dilute phases at a critical temperature (*T*_dem_), followed by a hardening transition via localized vitrification ($T_{\text{hard}}^{\text{PS}}$). We demonstrate that *T*_dem_ is governed by a mechanical-thermodynamic competition: The internal pressure (*P*_c_) to trigger demixing scales linearly with the permanent-network shear modulus (*G*_x_), maintaining a ratio of *P*_c_/*G*_x_ ≈ 100. We identify *T*_hard_ and $T_{\text{hard}}^{\text{PS}}$ as distinct hardening points, with elastically constrained local water content governing $T_{\text{hard}}^{\text{PS}}$. This framework provides a strategy for programming nonequilibrium fates and “lockable” properties of responsive soft matter.

## INTRODUCTION

Hydrogels serve as vital synthetic models for studying the nonequilibrium processes inherent to living matter, such as adaptation and self-organization (*1*, *2*). While conventional hydrogels typically consist of highly swollen networks (>70 wt % water), “crowded” hydrogels incorporate an abundance of associative bonds. These reversible cross-links suppress swelling and maintain low equilibrium water contents (<60 wt %), imparting unique viscoelasticity, self-healing capacity, and exceptional toughness (*3*–*7*). Crucially, these hydrogels exhibit distinct nonequilibrium thermal responses under rapid thermal stimuli, governed by the rate mismatch between heat conduction and the cooperative diffusion of the polymer network (*8*–*10*). Recent studies on crowded hydrogels, such as chemically cross-linked polyacrylic acid complexed with calcium ions (PAAc-CaAc_2_), have revealed heating-induced microphase separation arising from the strengthening of entropy-driven associative bonds (*11*, *12*). Rapid heating instantly drives the gel into a supersaturated state where the excess chemical potential of trapped water manifests as an internal pressure, triggering demixing instability within the crowded network.

In simple polymer solutions, demixing instability typically leads to spontaneous phase separation followed by coarsening. In hydrogels, however, this evolution is fundamentally constrained by the permanently cross-linked covalent network. The coupling of local concentration fluctuations to network deformation imposes an elastic energy penalty that opposes the driving force of water supersaturation, thereby elevating the demixing threshold (*13*, *14*). Furthermore, the interplay between network elasticity and interfacial energy suppresses coarsening, arresting the morphology at micro- to nanoscales (*15*, *16*). Because hydrogels are open systems, this confined phase separation represents a transient, nonequilibrium response; over extended timescales, the gel inevitably relaxes toward thermodynamic equilibrium through spatially homogeneous volume shrinkage (*10*, *17*, *18*).

In crowded hydrogels, water plays a dual role: It provides the thermodynamic driving force for phase separation while simultaneously acting as a plasticizer that regulates polymer segmental mobility. Consequently, dehydration elevates the glass transition temperature (*T*_g_), suggesting that vitrification can kinetically arrest, or “lock” the system’s structural evolution (*19*). In simple polymer solutions, this kinetic arrest is classically described by the Berghmans point, i.e., the intersection of the binodal boundary with the glass transition curve (*20*, *21*). In permanently cross-linked hydrogels, however, the landscape is fundamentally more complex. Network elasticity does not merely govern the onset and morphology of phase separation, but also couples with vitrification by constraining local water distribution and polymer density. Despite its importance for designing “smart” structural materials, the multiscale interplay between network elasticity, phase separation, and vitrification has not been systematically explored.

In this paper, we establish a physical framework in which the network elasticity acts as the governing constraint that regulates confined phase separation and dehydration-driven vitrification, leading to the counterintuitive phenomenon of thermally induced hardening. We investigate two divergent heating trajectories (Fig. 1A): a quasi-isobaric pathway (path A), which allows for water exchange with the surrounding environment, and a quasi-isochoric pathway (path B), where the water is kinetically trapped within the network. Along path A, the thermodynamic driving force remains insufficient to overcome network elasticity; consequently, the hydrogel contracts homogeneously until it hardens through global vitrification at a transition temperature defined as *T*_hard_. In contrast, rapid heating along path B induces a supersaturated state that overcomes the elastic constraint, triggering microphase separation at *T*_dem_. At higher temperatures, localized vitrification of the resulting polymer-dense phase governs the overall soft-to-stiff transition, defining a second characteristic temperature, $T_{\text{hard}}^{\text{PS}}$.

**Fig. 1.**
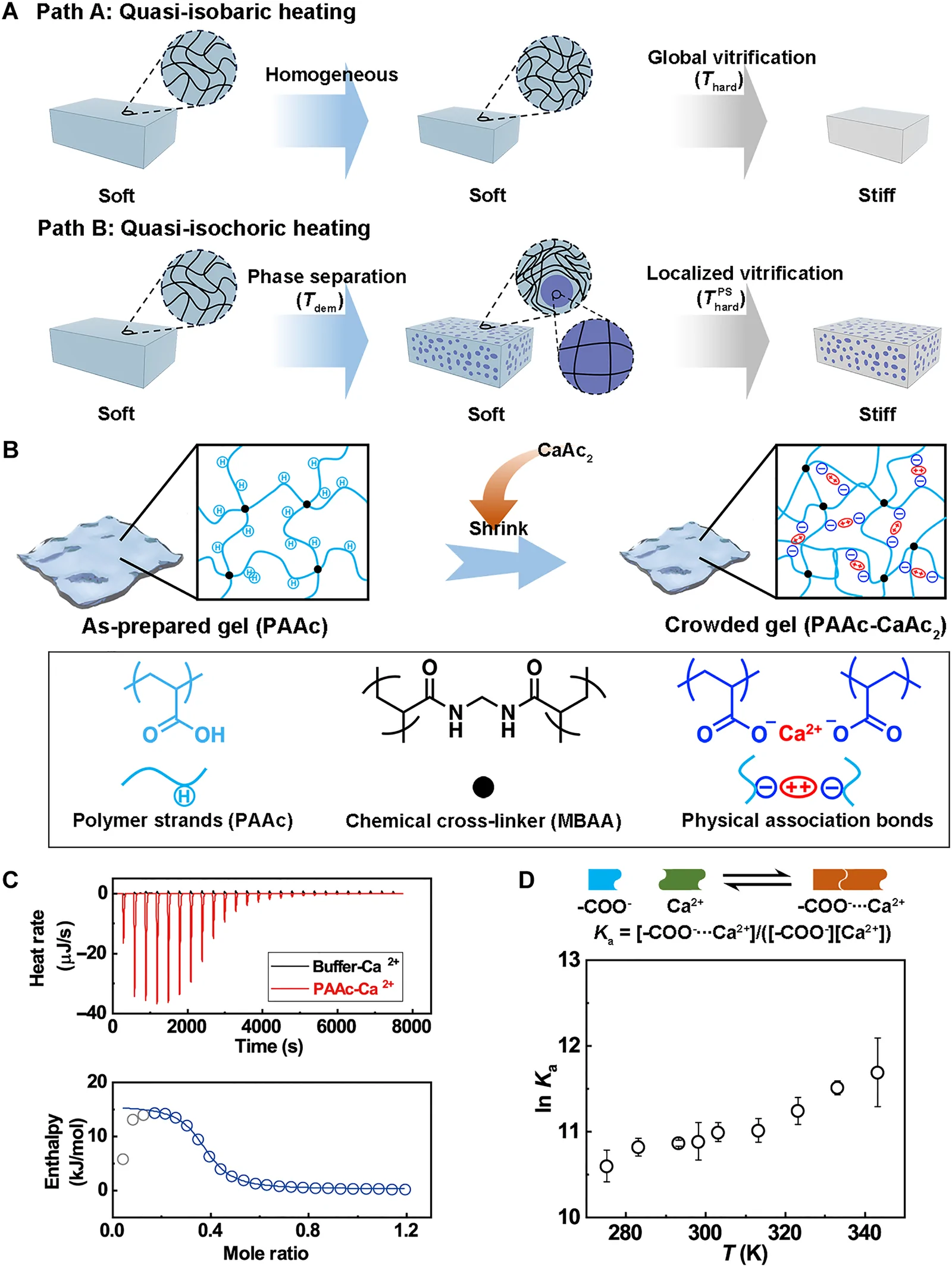
The entropy-driven crowded gel system and experimental design. (**A**) Illustration of two distinct heating pathways and the corresponding evolutionary fates of the hydrogel during heating. Path A: Slow heating induces a quasi-isobaric volume-shrinkage evolution, leading to a homogeneous hardening state by global vitrification. Path B: Rapid heating induces a quasi-isochoric evolution, resulting in a phase-separated hardening state through localized vitrification. (**B**) Preparation of the crowded hydrogel system (PAAc-CaAc_2_). An as-prepared PAAc hydrogel, chemically cross-linked by MBAA, is equilibrated in a CaAc_2_ solution. The formation of PAAc-Ca^2+^ coordination complexes triggers macroscopic shrinkage toward a thermodynamic equilibrium state. These reversible associative bonds act as an entropy-driven “molecular motor” that drives thermal responsiveness, while the permanent chemical cross-links provide the elastic “governor” that regulates the kinetics and morphology of the response. (**C**) Representative ITC results at 25°C, corresponding to fig. S5. Top: Baseline-corrected heat flow with time (black, background signal from CaCl_2_ titration into borate buffer; red, the complexation between Ca^2+^ and linear PAAc using borate buffer; pH 9.02). Bottom: Background-subtracted enthalpy per injection and its fitting curve. Mole ratio is defined as the concentration ratio of added Ca^2+^ to total PAAc repeat units. The results indicate that the complexation between Ca^2+^ and PAAc is an endothermic process. (**D**) Temperature dependence of the association constant *K*_a_ for PAAc-Ca^2+^ complexation, determined via ITC in dilute solution (borate buffer, pH 9.02). The increase in *K*_a_ with temperature reflects the intensifying driving force for network contraction and water expulsion upon heating.

To elucidate the underlying mechanism, we address three core questions: (i) How does the entropy-driven response of molecular associative bonds dictate macroscopic thermodynamic equilibrium and volume contraction? (ii) How does the hydrogel’s state evolution diverge between isobaric and isochoric pathways, and what distinct phase diagrams characterize these nonequilibrium fates? (iii) How does tuning the network elasticity modulate the demixing threshold, the resulting morphological scaling, and the final kinetic arrest temperature? Answering these questions provides a rational design strategy for “lockable” soft materials that leverage the competition between thermodynamics and elasticity to achieve high-performance functional properties (*22*, *23*).

## RESULTS

### The entropy-driven crowded gel system

#### The elastic framework and the crowded state

To investigate how permanent network constraints regulate nonequilibrium transitions, we synthesized a series of chemically cross-linked polyacrylic acid (PAAc) hydrogels with varying cross-linker densities (Fig. 1B). The concentration of cross-linker, *N*,*N*′-methylenebisacrylamide (MBAA), was varied from 0.01 to 5.0 mol% relative to the AAc monomer concentration (3 M). The “crowded” state was achieved by equilibrating these as-prepared gels in a fixed volume of 250 mM calcium acetate (CaAc_2_) solution. In this environment, divalent Ca^2+^ ions form an abundance of reversible associative bonds with the carboxylate groups on the polymer strands (fig. S1). This additional dynamic cross-linking drives pronounced macroscopic contraction, resulting in equilibrium volume ratios (*Q*_V_) of ≈0.4 to 0.5 relative to the as-prepared state (tables S1 and S2). Compared to the as-prepared PAAc hydrogels, the resulting crowded PAAc-CaAc_2_ hydrogels exhibit higher strength and toughness (fig. S3), attributed to the effective energy dissipation provided by these reversible associative bonds (*24*). Stress relaxation measurements demonstrate that the stress contribution associated with transient entanglements relaxes over short timescales under deformation (fig. S2). Consequently, the shear modulus arising specifically from the permanent cross-links in the PAAc-CaAc_2_ hydrogels (*G*_x_) serves as the “governor” for all subsequent nonequilibrium phenomena.

To isolate the contribution of this permanent network from the reversible associative bonds, *G*_x_ was derived from the permanent cross-linking component of the as-prepared PAAc modulus (*G*_x,0_) at room temperature (RT) and subsequently corrected for the volume change (*Q*_V_) occurring during the crowding process assuming an affine deformation of network strands (fig. S4 and table S2) (*25*–*28*).

#### The molecular driver: Entropy-driven association

While *G*_x_ provides mechanical resistance, the entropy-driven complexation between Ca^2+^ and carboxylate units provides the thermodynamic “motor” for thermal response. Using isothermal titration calorimetry (ITC) on a model system (linear PAAc and CaCl_2_), we quantified this interaction at 25°C, obtaining a binding constant *K*_a_ = (5.34 ± 1.17) × 10^4^ M^−1^ and a free energy change Δ*G* = −(26.9 ± 0.6) kJ/mol. The magnitude of Δ*G* corresponds to approximately 11 times the thermal energy (11*k*_B_*T*), indicating a strong and stable association (Fig. 1C). Thermodynamic decomposition reveals that the process is entirely entropy-dominated, with positive changes in both enthalpy (Δ*H*) and entropy (Δ*S*) (fig. S5). Crucially, both Δ*H* and Δ*S* increase with temperature (fig. S6 and table S3), yielding a positive heat-capacity change [Δ*C*_p_ = (0.20 ± 0.01) kJ/(mol·K)] (fig. S7). This signature is a hallmark of the release of structured water molecules from the hydration shells of the Ca^2+^ ions and polymer strands upon bond association (*29*, *30*).

This entropy-driven mechanism results in the thermally enhanced association (Fig. 1D and table S3): As temperature rises, the entropic contribution (*T*Δ*S*) increases, causing the binding free energy Δ*G* (Δ*G* = −*RT*ln *K*_a_) to become more negative. Consequently, heating generates a chemical potential gradient that drives the gel to contract further. The eventual macroscopic state is determined by the competition between this dehydration-driven contraction and the elasticity of the permanent covalent network.

### Path A: Quasi-static heating and the homogeneous hardening transition

#### Thermodynamic equilibrium and kinetic arrest

To establish the thermodynamic limit of the PAAc-CaAc_2_ system, we first applied a quasi-static heating protocol (0.1°C/hour) to a sample with a thickness of ≈1.2 mm at 0°C, termed the isobaric pathway. Under these conditions, the heating rate is sufficiently slow to allow water to diffuse out of the gel, maintaining chemical potential equilibrium with the surrounding bath.

For a representative gel (*c*_MBAA_ = 1.0 mol%, *G*_x_ = 67.8 kPa at RT), optical transparency is maintained throughout heating from 0° to 70°C (Fig. 2A), indicating a spatially homogeneous state. Below 50°C, the gel undergoes continuous, reversible volume contraction by expelling water (Fig. 2B, top, and table S4). In this regime, the volume decreases linearly with the increase in the association constant *K*_a_ (Fig. 2C), suggesting that the macroscopic volume is primarily controlled by the fraction of associative bonds at equilibrium. We denote the volume and temperature in this thermodynamic equilibrium state as *V*_eq_ and *T*_eq_, respectively. However, above 50°C, the contraction rate slows markedly, and the volume becomes nearly independent of both temperature and *K*_a_ [Fig. 2, B (top) and C]. We attribute this kinetic arrest to water loss, which reduces plasticization and raises the glass transition temperature (*T*_g_) of the polymer network. As the network dehydrates, the polymer chains eventually “freeze” into a glassy state, inhibiting the cooperative segmental motion required for further contraction and effectively “locking” the remaining water within the network (*31*).

**Fig. 2.**
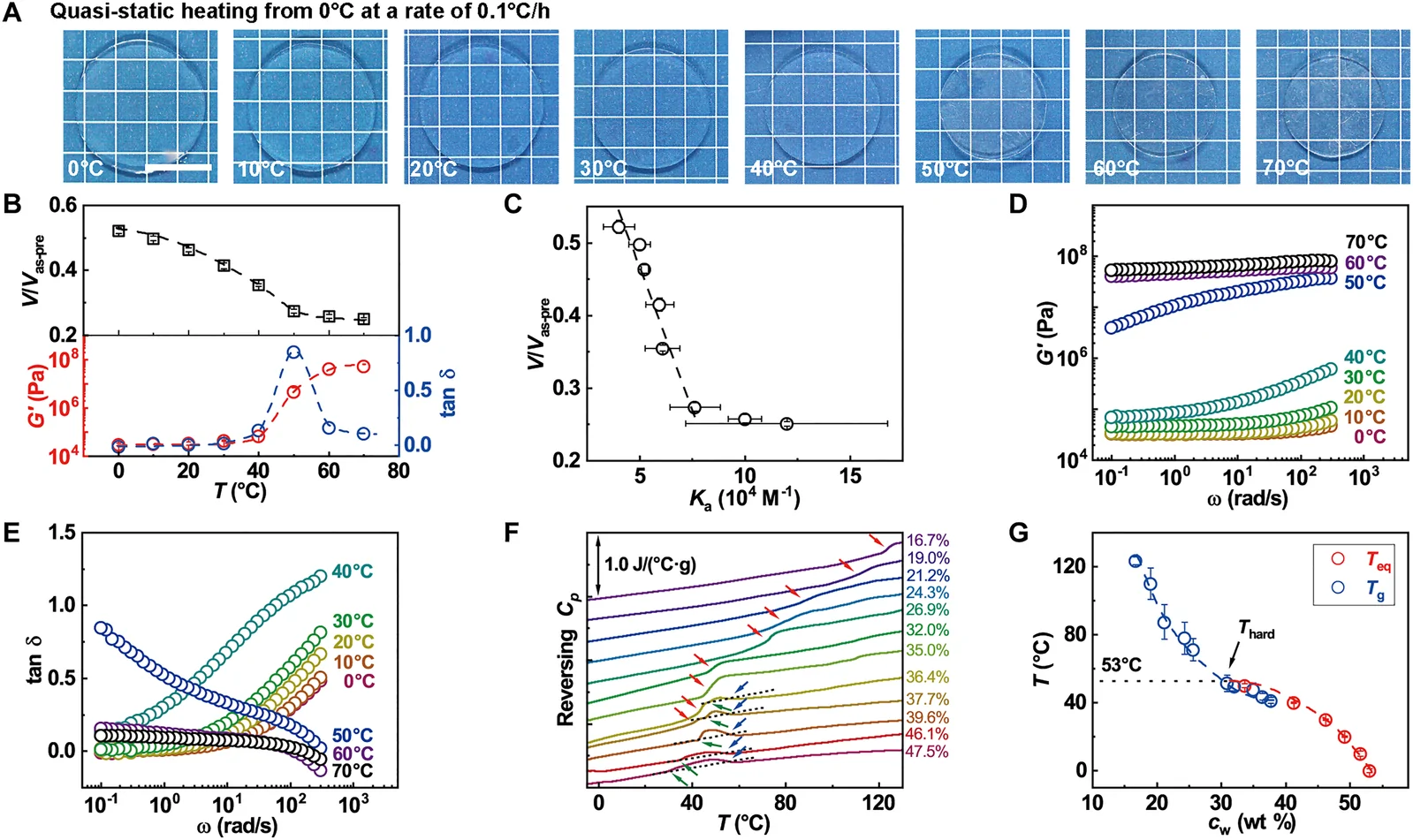
Thermodynamic properties and the homogeneous hardening transition along the quasi-isobaric pathway. (**A**) Sequential photographs during quasi-static heating. The gel maintains transparency throughout the pathway, indicating a homogeneous state. Scale bar, 1.0 cm. (**B**) Temperature dependence of *V/V*_as-pre_ (top) and *G*′ and tan δ at 0.1 rad/s (bottom). (**C**) Relationship between *K*_a_ and *V/V*_as-pre_. A linear relationship below 50°C suggests that the volume is governed by associative bonding equilibrium; the deviation above 50°C signals the onset of chain vitrification. (**D**) Storage modulus (*G*′) and (**E**) tan δ from frequency sweep tests. Measurements were taken after the gel reached a steady state at each temperature point along the quasi-isobaric pathway. (**F**) MDSC characterization of state transitions: Reversing heat capacity (*C*_p_) traces for gels with various water contents. Red arrows indicate *T*_g_ for samples before demixing. The black dotted lines represent the extrapolated baseline; deviations (green arrows) and drops below the baseline (blue arrows) signal the onset of phase separation and the subsequent hardening transition, respectively. The values on the right indicate *c*_w_ of hydrogels corresponding to each trace. No glass transition was detected for gels with >38 wt % water. (**G**) Temperature dependence of *c*_w,eq_ at equilibrium (red curve) and *T*_g_ as a function of *c*_w_ (blue curve). The intersection of these curves defines *T*_hard_. Note: A representative gel (1.0 mol% cross-linker, *G*_x_ = 67.8 kPa, ≈1.1 mm thick at RT) was used for measurements. Dashed lines in (B), (C), and (G) are guides for the eye. All measurements, except for *T*_g_, were repeated at least twice. Error bars for *T*_g_ span the onset and end temperatures of the glass transition.

#### Rheological validation of vitrification

To validate this mechanism, we performed time-temperature superposition rheological measurements on hydrogels with two fixed water contents (*c*_w_) (fig. S8). Both samples exhibit classical thermo-rheological behavior. Specifically, the sample with lower water content (*c*_w_ = 41 wt %) displays a tan δ peak within the experimental frequency (ω) window, whereas for the sample with higher water content (*c*_w_ = 46 wt %), this peak shifts toward the high-frequency end. This trend confirms that the observed thermal hardening is a direct result of dehydration-induced increases in *T*_g_, which slows segmental relaxation of the polymer network. Subsequently, we mapped the mechanical evolution along the isobaric pathway by performing frequency sweeps on hydrogels that had reached a steady state at different temperatures (Fig. 2, D and E, and fig. S9). The progression follows three distinct mechanical regimes. (i) Rubbery state (*T* < 40°C, *c*_w_ > 45 wt %): The storage modulus *G*′ remains low (∼10^5^ Pa) and nearly frequency independent. (ii) Viscoelastic state (*T* = 40 to 50°C, *c*_w_ = 30 to 45 wt %): Progressive dehydration restricts polymer mobility, causing *G*′ to rise and develop a pronounced frequency dependence as the system approaches the glass transition. (iii) Glassy state (*T* > 50°C, *c*_w_ < 30 wt %): The gel enters a glassy regime characterized by a sharp surge in *G*′ to values exceeding 10^7^ Pa. By identifying the characteristic peak in the loss factor (tan δ) at low frequency (0.1 rad/s) (Fig. 2B, bottom), we define the homogeneous hardening temperature (*T*_hard_) at approximately 50°C.

The *T*_hard_ determined from rheology coincides closely with the temperature marking the rapid surge in *G*′ and the cessation of macroscopic shrinkage (Fig. 2B). Below *T*_hard_, the hydrogel remains in a rubbery state, capable of reaching thermodynamic equilibrium with the surrounding solution. Above *T*_hard_, however, vitrification arrests chain relaxation and network deformation. Consequently, the thermodynamic driving force can no longer trigger net water expulsion or further contraction on the experimental timescale.

These results indicate that, at a fixed water content, thermally enhanced associative interactions do not appreciably alter the mechanical response of the hydrogel. Instead, the dominant factor governing the mechanical properties is the water content itself, which directly controls segmental relaxation. Therefore, the primary role of associative bonding in path A is thermodynamic rather than structural: It acts as the driving force that shifts the equilibrium water content upon heating, thereby indirectly driving macroscopic contraction and subsequent global vitrification. This confirms that the hardening in path A is a plasticization-controlled process dictated by water loss, rather than a direct result of increased physical association density.

Physically, *T*_hard_ represents the intersection of the thermodynamic equilibrium curve, *T*_eq_ versus *c*_w,eq_, and the water-dependent glass transition curve, *T*_g_ versus *c*_w_. Here, *c*_w,eq_ denotes the equilibrium water content at a given *T*, whereas *c*_w_ represents the general water content of the gel. To pinpoint this transition, we mapped the *T*_g_ across varying *c*_w_ using modulated differential scanning calorimetry (MDSC) (Fig. 2F and fig. S10) (*32*, *33*). The data show that *T*_g_ decreases as *c*_w_ increases, eventually becoming undetectable above a water content of around 38 wt %. This threshold aligns with our rheological results, suggesting that above this hydration level, the polymer chains remain sufficiently mobile to avoid vitrification even down to the freezing point of the solution. The crossover between the *T*_eq_-*c*_w,eq_ curve and the *T*_g_-*c*_w_ curve identifies *T*_hard_ at around 53°C (Fig. 2G). As this temperature is determined graphically from the intersection of two curves, it should be regarded as an approximate reference. Notably, it shows close agreement with the tan δ peak observed in our rheological tests (Fig. 2B).

It is important to note that above *T*_hard_, the storage modulus (10^7^ to 10^8^ Pa) remains one to two orders of magnitude lower than that of a fully dry glassy polymer (>10^9^ Pa). Therefore, “vitrification” in this context refers to the kinetic freezing of polymer segments on the experimental timescale rather than the attainment of a pristine, solvent-free glassy state. This discrepancy suggests that even in the vitrified state, the residual water continues to provide a degree of local plasticization within the polymer network. Crucially, this localized plasticization by the remaining solvent is known to enhance energy dissipation and prevent brittle failure, providing the gel with substantially higher toughness compared to its fully dehydrated counterpart (*34*). By maintaining this “moist” glassy state, the material avoids the inherent fragility of dry polymers while still achieving the structural rigidity required for high-performance applications.

### Path B: Rapid heating and confined phase separation

#### The quasi-isochoric state and internal pressure

When the hydrogel is heated rapidly, the rate of increase in associative bonding, reflected by *K*_a_, far outpaces the rate of net water transport out of the network. Because the water content (*c*_w_) remains effectively fixed on this short timescale, the gel enters a quasi-isochoric state. In this regime, the thermal enhancement of associations creates a water-supersaturated state. The excess chemical potential of trapped water manifests as an internal thermodynamic pressure, which acts as the driving force for demixing. Unlike the homogeneous contraction in path A, this trapped energy must be dissipated locally, triggering a transition to a phase-separated morphology within the constraints of the permanent covalent network.

To investigate path B, we used a rapid heating protocol (heating rates ≥1°C/min) to a representative sample (*c*_MBAA_ = 1.0 mol%, *G*_x_ = 67.8 kPa, ≈1.1 mm thick at RT). Under these conditions, the heating rate is sufficiently fast to maintain a constant macroscopic volume throughout the process (fig. S11). Above a critical temperature, the gel becomes turbid, signaling confined phase separation [Fig. 3, A (top row) and B]. We define this transition as the demixing temperature (*T*_dem_), corresponding to the threshold at which the entropy-driven internal pressure eventually overcomes the elastic constraint of the permanent network, as identified experimentally by the onset of turbidity in the gel (fig. S12).

**Fig. 3.**
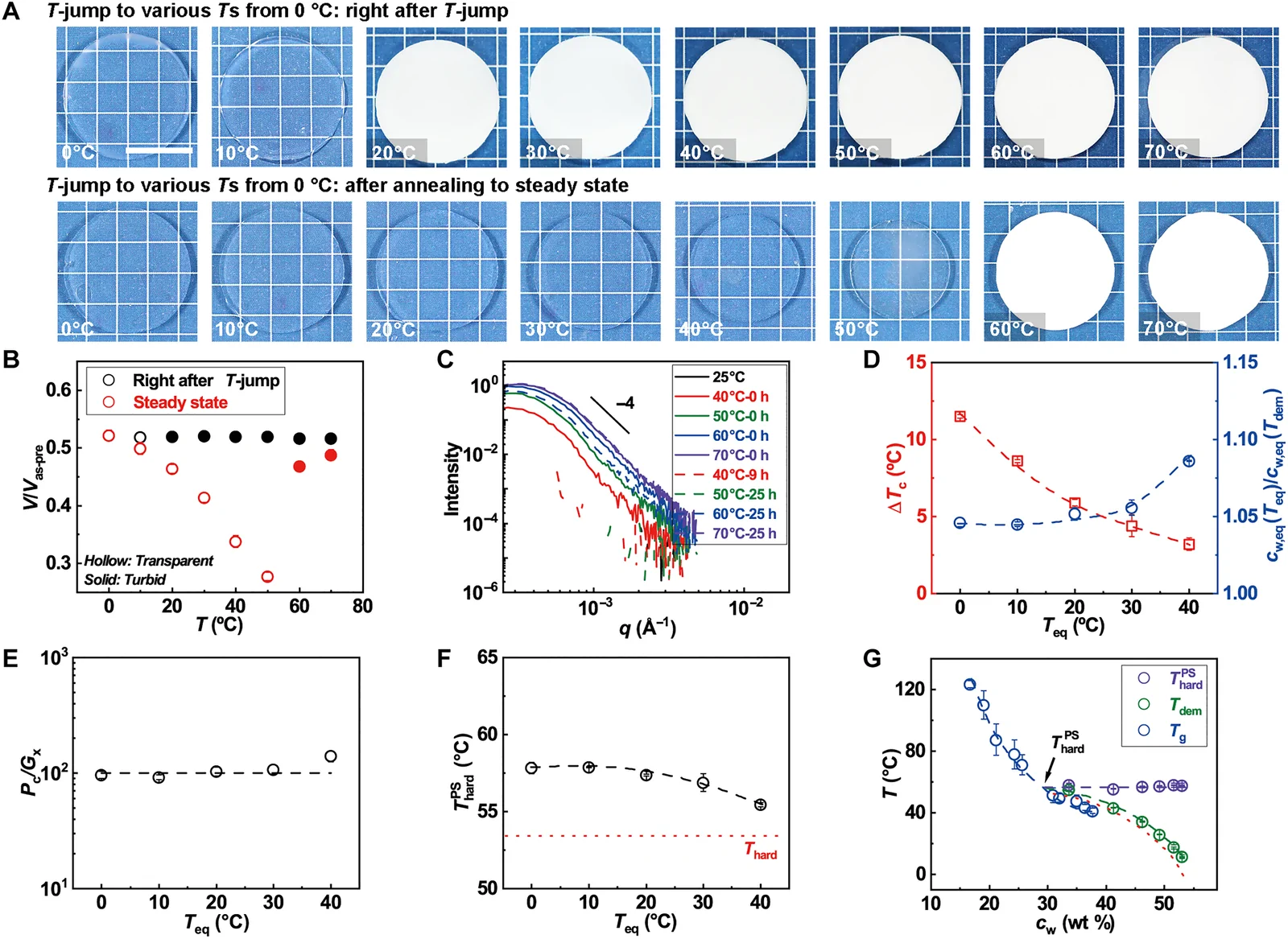
Kinetically driven confined phase separation and thermal hardening under quasi-isochoric conditions. (**A** and **B**) Evolution from a *T*_eq_ = 0°C initial state: (A) Time-lapse images following *T*-jumps to various temperatures. Top row: Immediate state after a quasi-isochoric *T*-jump. Bottom row: Reaching the steady state after long-time annealing. Scale bar, 1.0 cm. (B) Volume ratio *V*/*V*_as-pre_ shows the constant-volume regime during rapid heating (black circles) and the steady volume after annealing (red circles). Hollow and solid circles denote transparent and turbid states, respectively. (**C**) In situ USANS profiles. Solid curves represent the initial phase-separated state following the *T*-jump; dashed curves show the recovery to the steady state after long-time annealing. The scattering from the homogeneous states (25°C, 40°C-9 hours, and 50°C-25 hours) is close to the background level and therefore barely visible. (**D** to **F**) Influence of the starting *T*_eq_: (D) Minimum *T*-jump amplitude Δ*T*_c_ = *T*_dem_ − *T*_eq_ (left axis) required to trigger demixing, and the corresponding water supersaturation ratio *c*_w,eq_(*T*_eq_)/*c*_w,eq_(*T*_dem_) (right axis) as a function of *T*_eq_. (E) Correlation between *T*_eq_ and the critical internal pressure *P*_c_. *P*_c_ is normalized by the network’s shear modulus *G*_x_ at the corresponding *T*_dem_, yielding a constant ratio of approximately 100 across the rubbery regime. (F) $T_{\text{hard}}^{\text{PS}}$ determined from rheological measurements, with a weak dependence on *T*_eq_. The dotted line represents *T*_hard_ as determined graphically from Fig. 2G. (**G**) The *T*-*c*_w_ state space under quasi-isochoric conditions. The red dotted curve represents the isobaric equilibrium state (path A). Green and purple circles denote the demixing (*T*_dem_-*c*_w_) and phase-separated hardening ($T_{\text{hard}}^{\text{PS}}$-*c*_w_) transitions, respectively, both occurring under trapped-solvent conditions. The blue dashed curve represents the *T*_g_-*c*_w_ curve. The dashed lines in (D) to (G) are guides for the eye. Note: The same gel as in Fig. 2 was used for all measurements.

To visualize the architecture above *T*_dem_, we first used scanning electron microscopy (SEM). The SEM images reveal a well-defined porous structure at the microscale (fig. S13 and table S5). While the pore radius (*R*_SEM_ ≈ 1.6 μm) remains essentially constant across different temperatures, the pore density increases with rising temperature. Recognizing that sample dehydration for SEM observation can introduce artifacts, potentially exaggerating pore sizes, we performed ultra-small-angle neutron scattering (USANS) to capture the true in situ state. The scattering intensity increases upon heating above *T*_dem_ (Fig. 3C and fig. S14), exhibiting a slope of −4 in the Porod region, confirming sharp interfaces between the phase domains. Guinier analysis reveals polymer-dilute domains with a characteristic radius *R*_SANS_ ≈ 0.5 μm (table S5). In agreement with our SEM observations, the characteristic domain size remains stable while the number density of these domains increases with temperature, as evidenced by the increased scattering intensity at higher temperatures. Crucially, this phase separation process is fully reversible and causes no permanent damage to the covalent network (fig. S15). The measured domain radius (*R*_SANS_ ≈ 0.5 μm) is an order of magnitude larger than the contour length of polymer strands (*L*_c_ ≈ 18 nm). Despite this notable localized expansion, the network remains intact (table S6). This leads us to conclude that the excess water accumulates into over-swollen, polymer-dilute domains rather than pure solvent droplets as has been reported in polydimethylsiloxane (PDMS)–based systems (*35*, *36*). The degree of over-swelling within these micro-compartments is ultimately governed by the finite extensibility of the local polymer network, which acts as a mechanical limit on the phase separation process.

The system’s ability to expel this trapped water depends on the vitrification state of the polymer-dense phase. When held isothermally in the rubbery state (below 50°C), the excess water eventually diffuses out and the gel returns to its transparent, equilibrium state with a volume of *V*_eq_ over an extended period. USANS profiles confirm this structural recovery, with the scattering intensity returning to the baseline level of the homogeneous state (Fig. 3C). In contrast, when the gel is held isothermally above 60°C, both the volume and the phase-separated morphology remain virtually unchanged [Fig. 3, A (bottom row) and B]. The USANS profiles retain a constant, high scattering intensity (Fig. 3C). This confirms that heating above a critical threshold triggers vitrification-induced kinetic freezing that “locks” the phase-separated structure.

#### *The predictive framework:* P*_c_/*G*_x_ scaling*

Because phase separation is triggered by water supersaturation, the demixing temperature (*T*_dem_) is intrinsically linked to the gel’s initial equilibrium state (*T*_eq_). Using rapid heating ramps (quasi–*T*-jumps) at a rate of 2°C/min to maintain quasi-isochoric conditions (fig. S16), we found that the critical quasi–*T*-jump amplitude (Δ*T*_c_ = *T*_dem_ − *T*_eq_) required to trigger demixing decreases as the starting *T*_eq_ increases (Fig. 3D). Consequently, for any given water content (*c*_w_), the *T*_dem_-*c*_w_ curve, representing the onset of phase separation, is shifted upward from the equilibrium *T*_eq_-*c*_w,eq_ curve by the magnitude of Δ*T*_c_ (Fig. 3G).

To quantify the driving force for this transition, we compared the equilibrium water contents at the initial temperature, *c*_w,eq_(*T*_eq_), with the equilibrium water content the gel would ideally have at the demixing point, *c*_w,eq_(*T*_dem_). Both values can be directly referenced to the temperature–water content (*T*_eq_-*c*_w,eq_) curve obtained under path A (quasi-isobaric conditions), as shown in Fig. 2G. The ratio *c*_w,eq_(*T*_eq_)/*c*_w,eq_(*T*_dem_), which serves as a quantitative measure of water supersaturation, was found to increase with *T*_eq_ (Fig. 3D). Assuming ideality, the resulting difference in the chemical potential of water, Δμ_w_, can be expressed as a critical apparent internal pressure (*P*_c_) using the molecular volume of water (*v*_w_)

$$\Delta \mu_{w}=k_{B}T_{\text{dem}}\text{ln}\left[\frac{c_{w,\text{eq}}\left(T_{\text{eq}}\right)}{c_{w,\text{eq}}\left(T_{\text{dem}}\right)}\right]=P_{c}v_{w}$$ (1)

This internal pressure, *P*_c_, triggers the volume-constrained demixing by overcoming the restorative force of the network (*14*). To evaluate this mechanical-thermodynamic competition, we calculated the ratio of *P*_c_ to the network’s permanent shear modulus (*G*_x_) at the demixing onset temperature (*T*_dem_). The values of *G*_x_ at *T*_dem_ were extrapolated from the RT measurements by accounting for both thermal energy changes and volume variations through affine scaling.

The ratio *P*_c_/*G*_x_ remains constant at approximately 100 for all starting *T*_eq_ below 40°C, independent of the starting *T*_eq_ (Fig. 3E). This constant ratio demonstrates that the onset of phase separation is fundamentally governed by a mechanical-thermodynamic competition: The internal pressure generated by water supersaturation must reach a specific multiple of the network’s elasticity to trigger binodal instability (*13*).

#### *Localized versus global vitrification–induced hardening* ($T_{\text{hard}}^{\mathrm{PS}}$ *versus T*_hard_)

The onset of phase separation at *T*_dem_ does not immediately alter the macroscopic mechanics (fig. S12). Instead, a second transition, phase-separated hardening ($T_{\text{hard}}^{\text{PS}}$), emerges as temperature rises further under isochoric heating. Experimentally defined by the tan δ peak in *T*-sweep rheological measurements, $T_{\text{hard}}^{\text{PS}}$ represents a distinct structural fate compared to the homogeneous hardening temperature (*T*_hard_ ≈ 53°C) observed in path A (Fig. 3F and fig. S17). For a gel with *G*_x_ = 67.8 kPa at RT, $T_{\text{hard}}^{\text{PS}}$ is notably several degrees higher than *T*_hard_.

The fundamental mechanism for hardening in path B is the same as that in path A: dehydration-induced elevation of *T*_g_. The distinction lies in how each pathway reaches the thermodynamic “intersection” that triggers vitrification: In path A, homogeneous hardening occurs at the intersection of the equilibrium *T*_eq_-*c*_w,eq_ curve and the *T*_g_-*c*_w_ curve (Fig. 2G). Here, the entire bulk system loses water to the environment until the homogeneous network vitrifies. In path B, however, phase-separated hardening occurs at the intersection of the demixing *T*_dem_-*c*_w_ boundary and the *T*_g_-*c*_w_ curve (Fig. 3G). In this pathway, quasi-isochoric heating first triggers microphase separation at *T*_dem_, forming polymer-dense and -dilute phases through internal water rearrangement, with vitrification of the polymer-dense phase governing the hardening transition. Because the permanent network imposes an elastic energy penalty, the polymer-dense phase cannot fully dehydrate to the equilibrated state; instead, its water content evolves along the demixing *T*_dem_-*c*_w_ boundary, while excess water is accommodated in the polymer-dilute phase. Upon reaching the *T*_g_-*c*_w_ curve, the evolving dense phase vitrifies and becomes kinetically locked. The resulting state corresponds to a supersaturated, kinetically frozen morphology.

Mechanistically, $T_{\text{hard}}^{\text{PS}}$ should remain nearly constant for hydrogels with the same elasticity but different initial water contents. Experimentally, however, it decreases modestly as the starting *T*_eq_ approaches *T*_hard_ (Fig. 3F). This weak dependence suggests that $T_{\text{hard}}^{\text{PS}}$ is likely governed by the kinetics of internal water redistribution. As vitrification is approached, polymer segmental mobility and cooperative diffusion become increasingly sluggish. At higher starting *T*_eq_ (lower overall water content), the polymer chains require less rearrangement before becoming kinetically arrested, resulting in a shorter time to vitrification and thus a lower $T_{\text{hard}}^{\text{PS}}$. Thus, the weak dependence on *T*_eq_ likely reflects a secondary kinetic effect associated with internal water redistribution.

Together, *T*_hard_ and $T_{\text{hard}}^{\text{PS}}$ represent two distinct Berghmans-like points in the context of polymer physics, i.e., intersections where a phase boundary meets the glass transition curve. In our system, *T*_hard_ is the Berghmans point for the homogeneous state reached via global dehydration and vitrification. In contrast, $T_{\text{hard}}^{\text{PS}}$ is the Berghmans point for the phase-separated state reached via internal water redistribution and localized vitrification of the polymer-dense matrix.

### Divergent phase landscapes: Quasi-isobaric versus quasi-isochoric trajectories

The thermal response of the PAAc-CaAc_2_ hydrogel can be summarized through two distinct phase diagrams representing the quasi-isobaric and quasi-isochoric heating pathways (Figs. 2G and 3G). Although both are constructed in *T*-*c*_w_ space, they describe fundamentally different physical states and capture divergent trajectories of the system’s evolution.

Under quasi-static heating (path A), the hydrogel maintains continuous solvent exchange with the environment, behaving as an open thermodynamic system. In this limit, the water content is not an independent variable; rather, it is uniquely determined by the equilibrium condition at each temperature. As shown in Fig. 2G, the hydrogel follows a single thermodynamic trajectory along the equilibrium *T*_eq_-*c*_w,eq_ curve. The system remains in thermodynamic equilibrium until this curve intersects the *T*_g_-*c*_w_ curve at *T*_hard_. Beyond this intersection, global dehydration and structural relaxation are kinetically arrested by the vitrification of the polymer network. In this landscape, metastable or phase-separated states are inaccessible in the long-time limit.

In contrast, rapid heating (path B) temporarily restricts solvent exchange, driving the gel into a quasi-isochoric state. Here, the total water content becomes effectively fixed, serving as an independent control parameter alongside temperature. Consequently, the state of the hydrogel is no longer “pinned” to the external environment but is jointly determined by its internal composition and thermal energy. Figure 3G illustrates the states accessible under these solvent-constrained conditions: Below *c*_w_ ≈ 30 wt %, the hydrogel is dominated by a single glass transition. Above *c*_w_ ≈ 30 wt % and *T* > *T*_g_, the fate of the gel depends on the temperature relative to $T_{\text{hard}}^{\text{PS}}$. Below $T_{\text{hard}}^{\text{PS}}$, polymer chains remain mobile, allowing the system to evolve toward an intrinsic equilibrium at a fixed *c*_w_. In this regime, the onset of phase separation is governed by the demixing curve. Above $T_{\text{hard}}^{\text{PS}}$, vitrification of the polymer-dense matrix arrests the morphology, preventing further evolution toward the global thermodynamic minimum.

The comparison between Figs. 2G and 3G reveals that hydrogels undergo a hierarchy of responses: rapid, short-time internal rearrangements (microphase separation) and slow, long-time global evolution (macroscopic shrinkage). Vitrification at low water content serves as the ultimate “lock,” suppressing relaxation and leading to distinct nonequilibrium fates determined by the heating rate and destination temperature. This framework further predicts that network elasticity should selectively regulate the quasi-isochoric landscape: Increasing elasticity should raise the energetic penalty for internal water redistribution, shift the demixing boundary, and consequently alter the temperature at which the phase-separated morphology becomes kinetically locked.

### The role of elasticity: Quantifying the mechanical penalty

As established, the permanent elasticity (*G*_x_) of the hydrogel acts as a “governor” that suppresses phase separation and influences the structure of the phase-separated domains (Fig. 4A). To quantify this, we investigated gels with varying cross-linker densities. Ultraviolet-visible (UV-vis) spectroscopy reveals that the transition to a turbid, phase-separated state becomes progressively less abrupt as *G*_x_ increases, particularly when heating from a low equilibrium temperature (fig. S18, A to E). Using a transmittance threshold of 95% to define the onset of *T*_dem_ (fig. S18F), we observed that both the critical *T*-jump amplitude Δ*T*_c_ and the water supersaturation ratio *c*_w,eq_(*T*_eq_)/*c*_w,eq_(*T*_dem_) increase with the modulus (Fig. 4B).

**Fig. 4.**
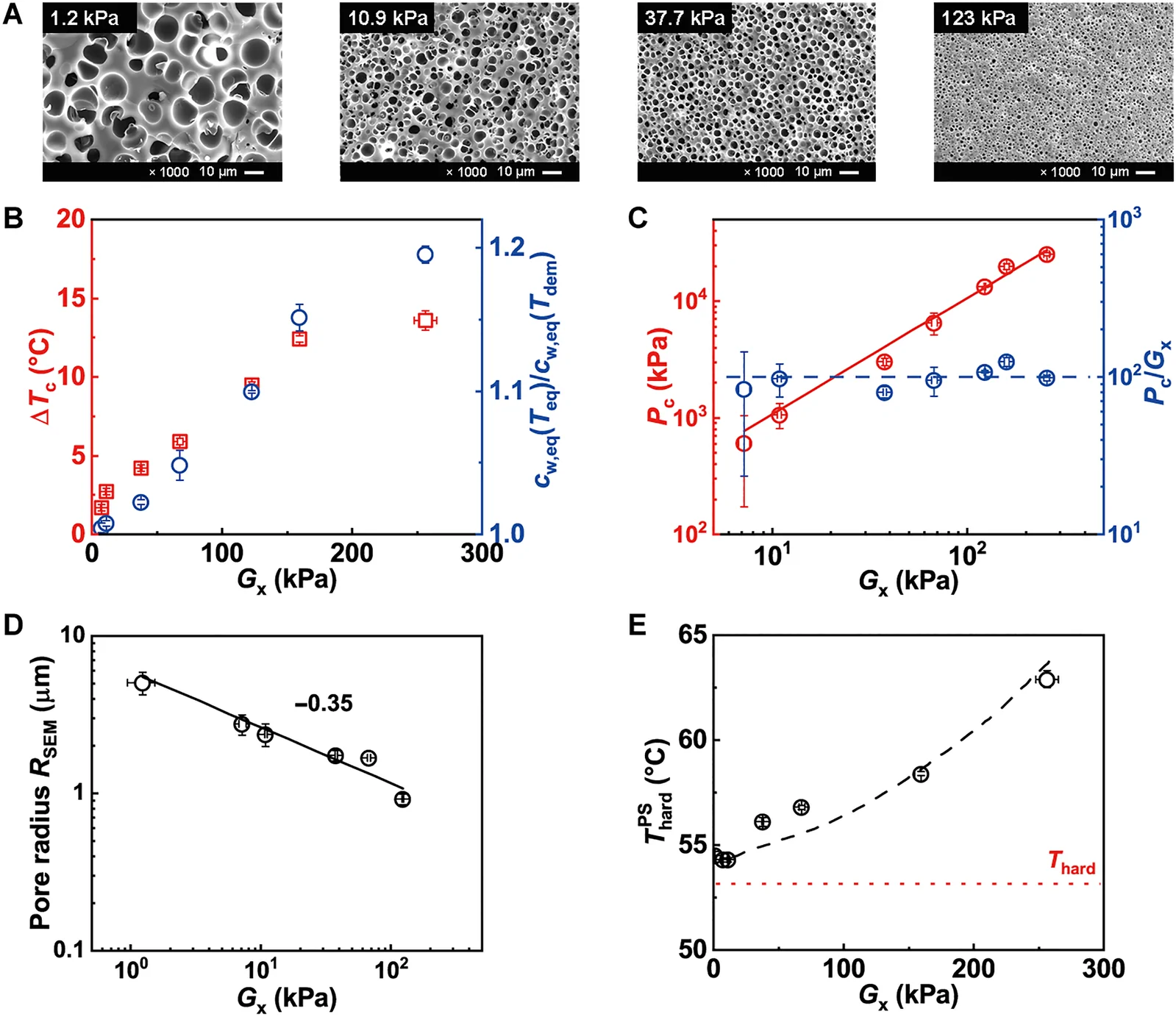
Effect of network elasticity on kinetically driven transitions. (**A**) SEM images showing the phase-separated microstructures of gels with different elasticities. Labels indicate *G*_x_ at room temperature. Gels were heated at 2°C/min from 20° to 80°C to vitrify the morphology before freeze-drying. Representative images are selected from fig. S19 and reproduced here for clarity. Scale bar, 10 μm. (**B**) The minimum *T*-jump amplitude Δ*T*_c_ = *T*_dem_ − *T*_eq_ (left axis) and the corresponding water supersaturation ratio *c*_w,eq_(*T*_eq_)/*c*_w,eq_(*T*_dem_) (right axis) as a function of *G*_x_. A higher cross-linker density imposes a greater elastic penalty, requiring higher water supersaturation to trigger demixing. (**C**) The critical internal pressure *P*_c_ (left axis) and the normalized ratio *P*_c_/*G*_x_ (right axis) as a function of *G*_x_. The solid line in the log-log plot represents a linear fit with a slope of unity, showing that *P*_c_ scales linearly with *G*_x_. The ratio *P*_c_/*G*_x_ remains constant at ≈100 over the investigated *G*_x_ range, identifying the mechanical threshold for confined phase separation. (**D**) Relationship between the pore radius *R*_SEM_ (derived from SEM analysis) and *G*_x_. The solid line in the log-log plot yields a slope of −0.35, close to the theoretical scaling prediction (*R* ∝ *G*_x_^−1/3^) for elastically arrested phase separation (*15*). (**E**) $T_{\text{hard}}^{\text{PS}}$ obtained from *T*-sweep rheological measurements as a function of *G*_x_. The red dotted line marks *T*_hard_, estimated graphically from the equilibrium phase diagram, which remains nearly independent of *G*_x_ within the studied range. In contrast, $T_{\text{hard}}^{\text{PS}}$ increases with *G*_x_. Consequently, the two hardening temperatures converge as the elastic constraint decreases. All gels were initially equilibrated at 20°C (thickness: ≈0.8 mm). Dashed curves in (C) and (E) are guides for the eye. In (D), the error bars for *R*_SEM_ represent the statistical variation in pore size measured from SEM images.

Using Eq. 1, we calculated the critical internal pressure (*P*_c_) required to trigger phase separation at a fixed starting temperature (*T*_eq_ = 20°C). We found that *P*_c_ increases linearly with the network shear modulus *G*_x_, such that the ratio *P*_c_/*G*_x_ ≈ 100 remains constant over the investigated *G*_x_ range (Fig. 4C). This value is remarkably consistent with the results obtained from our representative gel (*c*_MBAA_ = 1.0 mol%, *G*_x_ = 67.8 kPa at RT) across different temperatures (Fig. 3E). For simplicity, we used *G*_x_ measured at RT instead of the modulus at the demixing temperatures, which introduced a negligible error (<5%).

The observed *P*_c_/*G*_x_ ≈ 100 is substantially higher than the values reported in the literature or predicted by classical theories. For instance, the cavitation model, which assumes the formation of pure liquid droplets, predicts a ratio of only 5/2 (*14*, *37*). Similarly, studies on cooling-induced demixing in PDMS-based gels report a *P*_c_/*E* ratio of ≈10, where *E* is the Young’s modulus of the dry PDMS elastomer (*38*). In PDMS gels, the over-swelling of the dilute phase during demixing typically induces fracture of the brittle covalent network, leading to a morphology of nearly pure liquid droplets within a damaged polymer matrix (*35*). In stark contrast, the high fracture toughness of PAAc-CaAc_2_ hydrogels, arising from the abundant reversible ionic bonds, prevents such phase separation–induced damage. This structural robustness ensures that the phase separation remains fully reversible, allowing the network to accommodate large localized strains without catastrophic failure. As a result, our system undergoes a binodal-type demixing into polymer-dense and polymer-dilute phases, analogous to lower critical solution temperature (LCST) behavior in polymer solutions but fundamentally modified by network elasticity. This higher *P*_c_/*G*_x_ ratio suggests that the elastic penalty does not arise merely from the resistance of the dense phase. Instead, the large stretching of network strands within the over-swollen, polymer-dilute domains generates a massive localized elastic constraint. This localized “stretch penalty” explains why such extreme internal pressures, far exceeding the macroscopic modulus, are required to drive phase separation in these crowded ionic hydrogels.

While USANS provides high-fidelity structural data, SEM offers a practical means to track trends in domain size as a function of *G*_x_. To minimize dehydration artifacts during sample preparation, samples were heated at a rate of 2°C/min from *T*_eq_ = 20° to 80°C, well above $T_{\text{hard}}^{\text{PS}}$, to vitrify and “lock” the microstructure before freeze-drying. Such a heating rate allows sufficient development of the polymer-dilute domains while maintaining the hydrogel under quasi-isochoric conditions. The resulting pore size (*R*_SEM_) decreases as *G*_x_ increases (Fig. 4A, fig. S19, and table S6), following a clear scaling relationship: *R*_SEM_ ∼ *G*_x_^−0.35^ (Fig. 4D). Although the absolute values of *R*_SEM_ are larger than the in situ domain sizes due to dehydration-induced polymer aggregation or even network rupture during SEM preparation, *R*_SEM_ decreases systematically with increasing elasticity. This trend is qualitatively consistent with previous experiments and theoretical models of elastic microphase separation, where the characteristic length scale is governed by the competition between interfacial and elastic energies (*15*, *38*, *39*). Specifically, interfacial energy promotes domain growth to reduce surface area, whereas elastic deformation favors smaller, multidomain structures to minimize network distortion.

Last, we examined how elasticity influences the hardening transitions across varying cross-linker densities. Within the investigated range, the glass transition curves (*T*_g_ versus *c*_w_) and the thermodynamic equilibrium trajectories (*T*_eq_ versus *c*_w,eq_) remain nearly unchanged as elasticity varies, indicating that the homogeneous hardening temperature *T*_hard_ is essentially independent of cross-linker density (figs. S20 and S21). In this regime, the equilibrium properties are dominated by ionic association rather than by the elastic contribution of the covalent network. In contrast, the demixing boundary (*T*_dem_ versus *c*_w_) shifts systematically to higher temperatures as *G*_x_ increases. Consequently, the intersection between the demixing curve and the glass transition curve, which defines $T_{\text{hard}}^{\text{PS}}$, moves to higher temperatures (figs. S20 and S21). The trend is directly confirmed by the rheological measurements (Fig. 4E and fig. S22). Such behavior validates the thermodynamic framework proposed in Fig. 3G: Higher elasticity raises the energetic penalty for network deformation during internal water redistribution, thereby allowing the system to sustain greater water supersaturation and delaying the onset of both demixing and localized vitrification. In the limit of vanishing elastic constraint (*G*_x_ → 0), the demixing boundary would approach the equilibrium curve, and *T*_hard_ and $T_{\text{hard}}^{\text{PS}}$ would be expected to coincide. These results demonstrate that by simply tuning the covalent cross-linker density, one can systematically control both the scale of the internal micro-compartments and the temperature at which the material kinetically locks into its hardened state.

## DISCUSSION

The synergy between the covalent elastic framework and the entropy-driven “molecular motor” creates a unique platform for controlling nonequilibrium states in soft matter. By mapping the competition between thermodynamic driving forces (water supersaturation) and mechanical resistance (network elasticity), we can draw several broader conclusions:

The observation of a constant ratio *P*_c_/*G*_x_ ≈ 100 establishes a characteristic scaling law for crowded ionic hydrogels, revealing a threshold higher than those found for PDMS systems or predicted by simple cavitation models. The structural robustness provided by the dynamic bonds ensures that phase separation remains fully reversible, allowing the network to accommodate large localized strains without catastrophic failure. Consequently, demixing in these “crowded” gels resembles binodal-type phase separation in polymer solutions, yet is fundamentally constrained by network elasticity. Our results suggest that the high stretching of polymer strands within the polymer-dilute domains imposes a massive elastic penalty. This “stretch penalty” dominates the resistance to phase separation—a contribution likely diminished in brittle gels where over-swelling leads to network fracture. This highlights that the extensibility of the network, not just its modulus, is a critical parameter in governing phase stability.

While most traditional stimuli-responsive hydrogels are designed to transition between equilibrium states, our work demonstrates how to bypass equilibrium to create kinetically arrested morphologies. By tuning the heating rate, one can navigate between two distinct fates: (i) path A: A homogeneous, transparent contracted state (*V*_eq_) suitable for soft optics or precision actuators; (ii) path B: A heterogeneous, opaque “hardened” state for applications requiring rapid mechanical reinforcement or structural color. Because $T_{\text{hard}}^{\text{PS}}$ can be shifted predictably by adjusting *G_x_*, these materials can be locked into a hardened state at specific operational temperatures. This framework allows for the rational design of “lockable” soft materials whose destination state can be programmed by navigating the competition between thermodynamic driving forces and elastic constraints, offering a additional degree of freedom in material design.

The entropy-driven release of structured water and the subsequent localized vitrification echo sophisticated thermal protection mechanisms found in nature, such as desiccation tolerance of tardigrades or the stabilization of certain stress-tolerant proteins (*40*–*42*). In these biological systems, vitrification serves to shield delicate structures from mechanical or thermal degradation. The PAAc-CaAc_2_ system provides a simplified, abiotic model to study these complex bio-mechanical transitions, offering insights into how synthetic materials might achieve the robust environmental resilience seen in nature.

## MATERIALS AND METHODS

### Materials

Acrylic acid (AAc), MBAA, 2-oxoglutaric acid (α-keto), calcium acetate monohydrate (CaAc_2_·H_2_O), sodium acetate (NaAc), heavy water (D_2_O), PAAc (*M*_w_ = 5000), and boric acid were purchased from FUJIFILM Wako Pure Chemical Co. (Japan). All the gel precursor and salt solutions were prepared with deionized water unless otherwise specified.

### Isothermal titration calorimetry

The thermodynamic parameters of complex formation between PAAc and CaCl_2_ were measured using a Nano ITC (TA Instruments, USA). The PAAc (*M*_w_ = 5000) solution with 2.0 mM repeat-unit concentration and CaCl_2_ solution of 8.0 mM were prepared in 5 mM borate buffer. The pH of both solutions was kept at 9.02 ± 0.01 at 25°C. At this pH, the carboxylic acid groups on the chains are fully ionized. PAAc-5k solution [1.0 ml (actually 0.95 ml)] was added to the cell. After that, the CaCl_2_ solution was titrated into the polymer solution at a stirring rate of 350 rpm at various temperatures. The CaCl_2_ was delivered in 25 injections of 10 μl (actually 9.89 μl) per injection. The time interval was 300 s. For the measurement at 25°C, an independently measured blank curve of CaCl_2_ solution titrated into borate buffer was subtracted. For measurements at other temperatures, the heat of the final injection, at which the binding sites were assumed to be saturated, was used to estimate the background heat and was subtracted. The fitting model was a simple independent binding model, assuming all the binding sites on chains bind independently to Ca^2+^. For thermodynamic analysis, the initial data points deviating from the sigmoidal binding profile were excluded to achieve reliable fitting. These early injections are attributed to Ca^2+^ binding in a monodentate coordination mode, which precedes the formation of the dominant complexation motif. Accordingly, fitting was performed starting from the maximum of the binding curve. The binding ratio *n*, enthalpy Δ*H*, entropy Δ*S*, and association constant *K*_a_ were obtained. For reference, a control titration was also performed by titrating 8.0 mM CaCl_2_ solution into 2.0 mM sodium acetate (NaAc) solution prepared in borate buffer at the same pH.

### Synthesis of PAAc-CaAc_2_ hydrogels

The preparation of PAAc-CaAc_2_ gel consisted of two steps. The first step was the polymerization of the PAAc gel. A mixed aqueous solution containing AAc (3 M), MBAA (1.0 mol% of AAc), and α-keto (0.1 mol% of AAc) was injected into a reaction cell produced by two glass plates (100 mm by 100 mm) separated by a silicone rubber spacer (1.5 mm thickness). Then, the cell was transferred to a glove box filled with argon gas and irradiated with UV light (wavelength, 365 nm; intensity, approximately 4 mW/cm^2^) for 8 hours to obtain the PAAc gel. The as-prepared PAAc gel was cut into pieces of 70 mm by 70 mm with a thickness of 1.5 mm. The second step was the complexation of as-prepared PAAc gel with CaAc_2_. Each piece of as-prepared PAAc gel was soaked in 200 ml of CaAc_2_ solution with a concentration of 250 mM. The volume ratio between as-prepared PAAc gel and CaAc_2_ solution was 1:27 (i.e., the mole ratio of monomer and Ca^2+^ was 1:2.3). The CaAc_2_ solution was prepared by dissolving the CaAc_2_·H_2_O in deionized water. The PAAc gel shrunk in CaAc_2_ solution as a result of ion complexation between carboxylic groups of PAAc and Ca^2+^. After 7 days, the PAAc-CaAc_2_ gel reached an equilibrium state with a size of around 55 mm by 55 mm by 1.1 mm at RT. The criterion for determining equilibrium was that the hydrogel was transparent and its volume no longer changed.

By varying the MBAA concentration from 0.01 to 5.0 mol%, we prepared the hydrogels with different elasticities. Because a high cross-linker concentration tends to cause inhomogeneous polymerization inside the hydrogel, a thinner 1-mm silicone spacer was adopted. The volume of the soaking CaAc_2_ solution was correspondingly reduced to 130 ml. The PAAc-CaAc_2_ gels with cross-linker densities below 0.5 mol% required longer time (14 to 28 days) to reach equilibrium. After equilibration at RT, the hydrogel thickness was 0.7 to 0.8 mm. The prepared PAAc-CaAc_2_ gels showed higher toughness than the as-prepared PAAc gels.

### Synthesis of PAAc-NaAc hydrogels

The PAAc-NaAc gel was also prepared. The preparation conditions were the same as those for the PAAc-CaAc_2_ hydrogel, except for the type of soaking salt. The soaking solution contained 750 mM NaAc. After equilibrium, the gel swelled to a large volume and exhibited weak mechanical properties.

### Equilibrium volume measurement

Disc-shaped gels with a diameter of 20 mm were soaked in petri dishes containing CaAc_2_ solutions. The petri dish was placed on the heating stage of a temperature-control chamber (Msa-Factory PG1506 chamber, PCC107FL controller, Japan). For the quasi-static pathway, the gels were slowly heated or cooled to the prescribed temperatures at a rate of 0.1°C/hour. For the *T*-jump heating pathway, the gels were abruptly heated and then annealed at prescribed temperatures for a long time to reach constant volume. Then, the gels were removed from CaAc_2_ solutions, and their diameters were recorded using a camera (Canon EOS 7D, Japan).

### Water content measurement

The water content of the PAAc/CaAc_2_ gel equilibrated at various temperatures (*T*) was determined by freeze-drying. First, gels were weighed using a balance (SHIMADZU AUY220, Japan). Then, the gels were freeze-dried using a freeze dryer with a −80°C cold trap (Advantage XL-70, VirTis freeze-dryer, USA). After freeze-drying for 1 week, the dry gels were weighed. The weight fraction of water, *c*_w_, was calculated by *c*_w_ = (*m*_wet_ − *m*_dry_)/*m*_wet_, where *m*_wet_ represents the weight of hydrogels before freeze-drying and *m*_dry_ is the weight of dried gels. The contribution of CaAc_2_ to the dry weight was neglected.

### Preparation of PAAc-CaAc_2_ hydrogels with different water content

First, we prepared a homemade airtight mold using two glass plates (100 mm by 100 mm) and a 5-mm-thick silicone spacer. The internal space of the mold was divided into many small units. Then, the gels equilibrated at 20°C were cut into many small disc-shaped pieces with diameter ≈ 3 mm. Each piece was weighed using a balance (SHIMADZU AUY220, Japan), then placed individually into separate compartments of the airtight mold. Every 6 hours, one piece was taken out and weighed. The water content of each gel piece was calculated from the water content of the gel equilibrated at 20°C (*c*_w,20_), the initial weight of the piece (*m*_0_), and the weight of the same piece before testing (*m*_x_): *c*_w,x_ = 1 − *m*_0_ × (1 − *c*_w,20_)/*m*_x_. Because each sample was subsequently used for MDSC measurements, the water content was determined from a single measurement.

### Attenuated total reflectance Fourier transform infrared spectroscopy

The gels were prepared using D_2_O instead of deionized water for both the precursor and calcium acetate solutions. Attenuated total reflectance Fourier transform infrared spectroscopy ATR-FTIR spectroscopy (FTIR 6600, JASCO, Japan) was performed. The wave number region was 1800 to 800 cm^−1^, with 64 scans and a resolution of 2 cm^−1^.

### Rheology measurement

The viscoelastic properties of the PAAc-CaAc_2_ gels were measured using an ARES G2 rheometer (TA Instruments, USA) with a parallel plate geometry. A disc-shaped gel with a diameter of 15 mm was affixed between an upper parallel plate and a lower U-shape plate cup using superglue. The CaAc_2_ solution was poured into the U-shape cup to immerse the gel. The top of the U-shape cup was sealed with silicone film to prevent water evaporation.

Frequency sweep tests were performed over an angular frequency of 0.1 to 300 rad/s at a strain of 0.1% for gels annealed at various temperatures. Temperature sweep tests were performed from 5° to 90°C with a heating rate of 3.0°C/min, a strain of 0.1%, and an angular frequency of 10 rad/s. Before testing, the hydrogel was equilibrated in solutions at different temperatures and then attached between the plates. Because water exchange was suppressed after the gel was fixed between the plates, the initial temperature (5°C) and the subsequent heating process were assumed not to substantially change the water content of the hydrogel. $T_{\text{hard}}^{\text{PS}}$ was obtained from the tan δ peak in the temperature sweep test.

Stress relaxation tests were performed at 20°C under a constant shear strain of 1.0%, which was within the linear viscoelastic regime. The relaxation modulus was recorded for 1 hour. The relaxation modulus at 0.1 s was taken as the initial modulus (*G*_0_) for normalization to minimize the influence of the finite strain-establishment time and instrumental response. The normalized relaxation modulus was expressed as *G*(*t*)/*G*_0_.

### Modulated differential scanning calorimetry

The glass transition temperature (*T*_g_) of the PAAc-CaAc_2_ gel was measured using a Discovery DSC2500 (TA Instruments, USA). The small disc-shaped gels with different water contents were encapsulated in an alodined aluminum hermetic pan using an identical empty pan as the reference. The weights of the sample pan and reference pan were equal within 0.1 mg. The instrument was purged with nitrogen at a flow rate of 300 ml/min during measurement. The measurement was performed in modulated conventional mode. The underlying heating rate was 3°C/min, modulated by a sinusoidal temperature oscillation with an amplitude of 0.64°C and a period of 40 s. The temperature range was −10° to 140°C. The reversing heat capacity curve was obtained from the reversing heat flow and the heating rate. *T*_g_ was defined from the onset of the step change in the reversing heat capacity curve. In addition, *T*_dem_ and $T_{\text{hard}}^{\text{PS}}$ can be estimated from the reversing heat capacity curve: *T*_dem_ corresponds to the deviation from the post-*T*_g_ baseline, whereas $T_{\text{hard}}^{\text{PS}}$ corresponds to the intersection between the reversing heat capacity curve (or reversing heat flow curve) and the extrapolated post-*T*_g_ baseline. However, in this paper, the values of *T*_dem_ and $T_{\text{hard}}^{\text{PS}}$ were adopted from UV-vis transmittance measurements and the rheological temperature sweep tests, respectively.

### Transmittance measurements

The transmittance of the PAAc-CaAc_2_ gel during heating was measured at a wavelength of 550 nm using a UV spectrophotometer (Shimadzu UV-1800, Japan). A rectangular gel was placed in a quartz cuvette filled with a CaAc_2_ solution, while an identical quartz cuvette filled with deionized water was used as reference. Nitrogen purging was used to prevent surface condensation on the cuvettes. After preannealing at the initial temperature for 10 min, the cuvettes were heated to 70°C at a constant rate of 2°C/min. The demixing temperature *T*_dem_ was defined as the temperature at which the transmittance decreased to 95%. This threshold was selected to minimize uncertainty arising from the broad temperature range over which transmittance decreases. Under this protocol, the characteristic timescale for the observable onset of phase separation is larger than 10 s (from 100% to 95% transmittance). Stress relaxation measurements (fig. S2) indicate that the majority of the transient modulus has already relaxed within this timescale. Therefore, the measured *T*_dem_ reflects the competition between internal pressure and the elasticity of the permanent network.

### Estimation of shear modulus from permanent networks

The shear modulus of PAAc-CaAc_2_ gels consists of two distinct components. The first is the permanent elasticity (*G*_x_), which arises from chemical cross-linkers and trapped topological entanglements. The second component stems from nonpermanent constraints (*G*_e_), including transient chain entanglements and physical associations mediated by Ca^2+^ coordination. The entanglement contribution (*G*_e_) can lose its elastic contribution in two distinct ways: first, deformation-induced disentanglement at large strains, as demonstrated by the Mooney-Rivlin relationship (fig. S4); and second, stress relaxation over long timescales, even at small deformations (fig. S2). Because only the permanent network constraints provide the restorative force governing long-timescale elasticity and the elastic penalty for phase separation, it is essential to isolate *G*_x_. To do so, we first measured the permanent network modulus (*G*_x,0_) of the as-prepared PAAc hydrogels. We then estimated *G*_x_ in the crowded state by accounting for the volume contraction of the gel using the affine scaling relationship *G*_x_ = *G*_x,0_ × *Q*_V_^−1/3^, where *Q*_V_ (<1) represents the volume shrinking ratio (*V*_eq_/*V*_as-pre_) after equilibration in the CaAc_2_ solution.

To obtain the permanent network modulus (*G*_x,0_), tensile tests were performed using a commercial tensile tester (Instron 5965, Instron Co.), equipped with a noncontact video extensometer AVE. As-prepared PAAc hydrogels (3 M monomer) with varying cross-linker densities were prepared in a glass mold with a thickness of 1.0 mm and cut into dumbbell-shaped specimens with a gauge length of 17 mm and a width of 4 mm. Tensile tests were conducted at a loading rate of 100 mm/min (corresponding to an initial strain rate of 0.14 s^−1^). All tensile tests were conducted in air at RT. The stretch ratio was determined from the change in distance between two marker dots placed within the gauge section. Each measurement was performed at least in triplicate to ensure reproducibility, and the average values were reported. The resulting nominal stress-strain data were replotted as Mooney-Rivlin plots. To isolate the contribution of the permanent cross-links, *G*_x,0_ was calculated by the intercept of the Mooney-Rivlin plot at the limit of infinite stretch (1/λ → 0).

### Scanning electron microscopy

The microstructures of the PAAc-CaAc_2_ gel were observed using an SEM instrument (JSM-6010LA, JEOL, Japan). First, the gels equilibrated at 20°C were soaked in CaAc_2_ solutions and heated to target temperatures at a rate of 2°C/min. Next, they were removed and immediately quenched between two brass blocks precooled in liquid nitrogen. After 2 min, the frozen samples were immersed in liquid nitrogen for further freezing. After that, the frozen samples were freeze-dried for 1 week using a freeze-dryer (Advantage XL-70, VirTis freeze-dryer, USA). The prepared dry samples were carefully fractured with tweezers. The internal cross sections of the samples were sputter coated with a gold/palladium mixture using an E-1010 ion sputter system (Hitachi, Japan) and observed using SEM with an accelerating voltage of 20 kV. The pore size was measured and analyzed using ImageJ software.

### Ultra-small-angle neutron scattering

The measurements were conducted at SANS-U of JRR-3, Japan Atomic Energy Agency, Tokai, Japan. The neutron wavelength was 7 Å, and the sample-to-detector length was 12 m. The area of the high-resolution detector was 64.5 × 64.5 cm^2^, with a position resolution of 5 × 5 mm^2^. The pixel size was 450 μm. Equilibrium hydrogels with a thickness of 0.78 mm were prepared in CaAc_2_ solutions at RT. Before measurement, the disc-shaped hydrogels were sealed in quartz cells and equilibrated at 20°C. Subsequently, four cells were placed in four separate sample chambers and *T*-jump heated to 40°, 50°, 60°, and 70°C, respectively. USANS scattering patterns were collected every 15 min. The USANS 2D patterns were reduced to 1D profiles using the reduction package Red2D (v.2.5.4) on a data analysis software Igor Pro. The 1D profiles were then normalized by measurement time and transmission. The cell scattering was subsequently subtracted from all profiles.
