# Supplementary material for: Kinetic-driven microphase separation and vitrification of crowded hydrogels with associative bonds
Source: Sci Adv. 2026 Oct 16;12(42):eaed8808. doi: 10.1126/sciadv.aed8808 (PMC13674517; doi:10.1126/sciadv.aed8808)
Supplement: Supplementary file 1 [file sciadv.aed8808_sm.pdf]

Science Advances
AAAS

Supplementary Materials for

Kinetic-driven microphase separation and vitrification of crowded hydrogels with associative bonds

Dong Shi *et al.*

Corresponding author: Takayuki Nonoyama, nonoyama@sci.hokudai.ac.jp;
Jian Ping Gong, gong@sci.hokudai.ac.jp

Sci. Adv. **12**, eaed8808 (2026)
DOI: 10.1126/sciadv.aed8808

This PDF file includes:

Supplementary Text
Figs. S1 to S23
Tables S1 to S6

Supplementary Text

USANS data analysis

After phase separation, the PAAc-$CaAc_2$ hydrogel exhibits strong scattering, which is markedly higher than that of the empty cell. The characteristic size of polymer sparse domains was determined from the Guinier region of the scattering profiles. In this region, the intensity, $I(q)$, follows the Guinier approximation:

$$I(q) = I_0 \exp\left(-\frac{q^2 R_g{}^2}{3}\right) \tag{S.1}$$

Where I_0 is the scattering intensity at $q = 0$, q is the scattering vector and R_g is the radius of gyration. By plotting ln $I(q)$ versus q^2 in the low-q range, $q = (2.5 \sim 8.0) \times 10^{-4}$ Å^{-1}, *i.e.*, Guinier plot, we performed a linear fit and estimated R_g from the slope. For spherical objects, the radius, R, can be estimated from the R_g by

$$R_g = \sqrt{3/5}R \tag{S.2}$$

Over-swollen domain size statistics in SEM images

As shown in **Fig. S23**, the sizes of *over-swollen domains* in SEM images were identified as pores. The pore sizes were quantified using *Image J* software. First, the images were converted to binary format, and the pores were identified using thresholding. The area of each pore was measured automatically. Since SEM observes the cross section of the sample, many pores may not be captured at their maximum cross-sectional area, and due to contrast limitations, numerous noise artifacts can appear. Therefore, a low cutoff value of 0.05 μm^2 was applied, and only the largest one-third of the detected pores were used for statistical analysis.

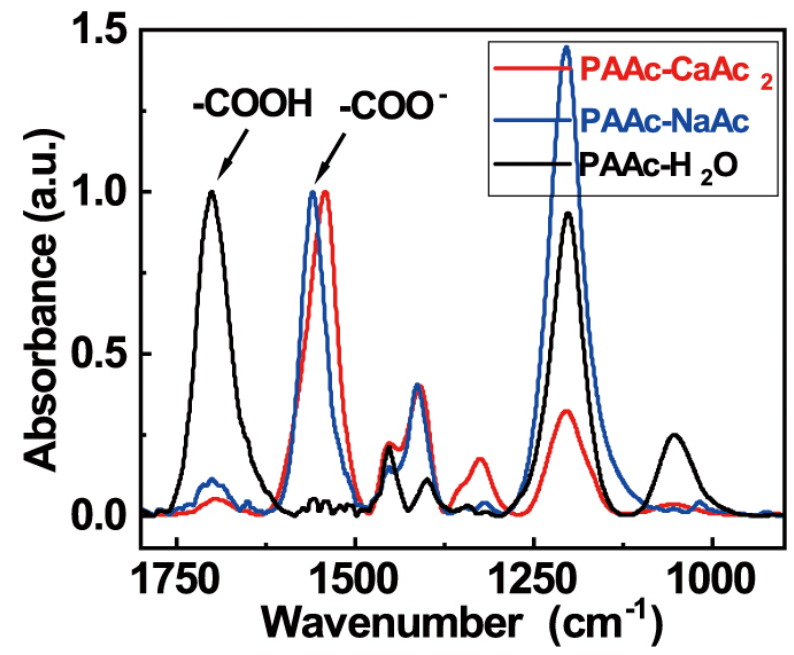

Fig. S1. ATR-FTIR spectra of hydrogels with 1.0 mol% cross-linker at *r.t.* The red and blue curves represent the absorbance of PAAc hydrogels equilibrated in $CaAc_2$ (250 mM) and NaAc (750 mM) solution, respectively. The black curve represents the absorbance of PAAc hydrogels equilibrated in pure water. During the preparation and equilibration, D_2O was used in place of deionized water in the solution. The peaks at 1700 cm^{-1} and 1540 cm^{-1} correspond to the stretching vibrations of carbonyl groups in -COOH and $-COO^-$ on the polymer chains, respectively. The spectra show that the carboxylic acid groups (-COOH) on PAAc fully dissociate into carboxylate ions ($-COO^-$) in both $CaAc_2$ and sodium acetate (NaAc) solutions. However, the gel swells markedly in NaAc solution ($Q_V > 1$) but shrinks in a $CaAc_2$ solution with the same ionic strength (Table S1). This indicates that the shrinkage in $CaAc_2$ is due to ionic bridging rather than electrostatic screening.

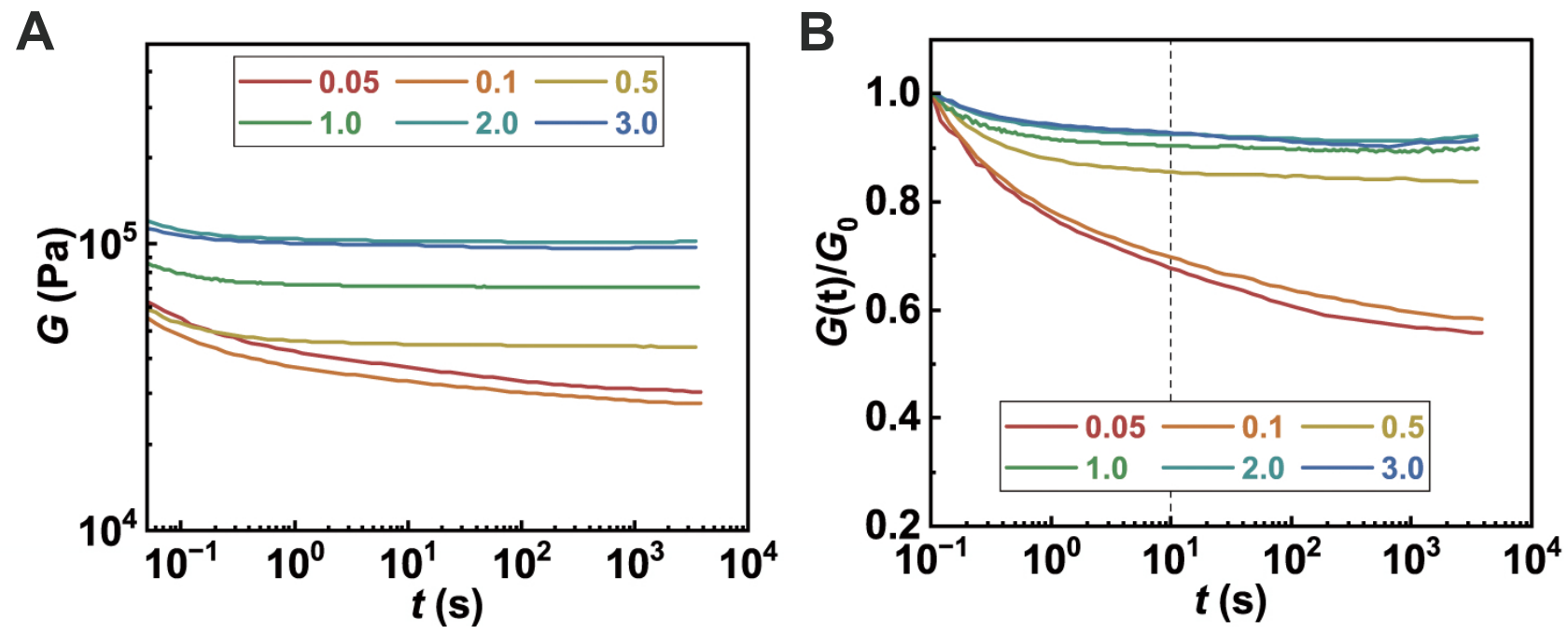

Fig. S2. Stress relaxation measurements of hydrogels with different cross-linking densities. (A) Relaxation modulus *G*(t) measured at 20 °C under a constant shear strain of 1.0% (within the linear viscoelastic regime) over a duration of 1 h; (B) Normalized relaxation modulus, $G(t)/G_0$, where G_0 is defined as the modulus at 0.1 s. The long-time plateau is predominantly governed by the permanent cross-linked network, whereas the decaying component corresponds to the transient contribution from chain entanglements. The dashed vertical line marks 10 s, corresponding to the characteristic timescale for determining the onset of phase separation under the heating protocol used in this study. By this timescale, hydrogels with $c_{\mathrm{MBAA}} \geq 0.5$ mol% have nearly reached their long-time plateau, whereas the more lightly cross-linked samples have already undergone substantial transient relaxation. The numbers within the figure indicate the cross-linker density in mol%.

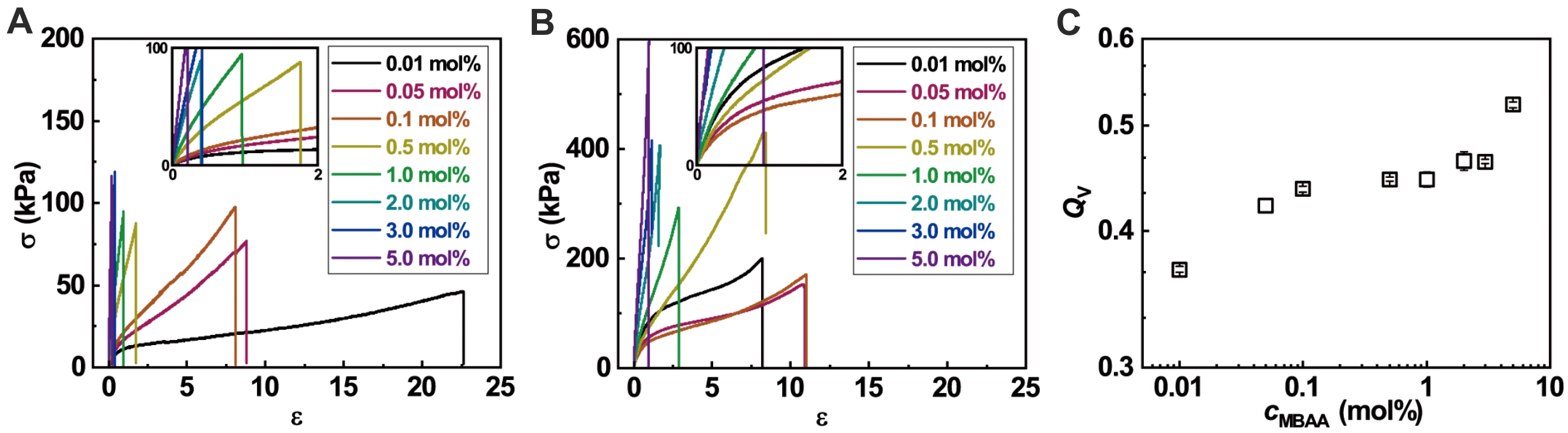

Fig. S3. Properties of as-prepared PAAc and equilibrated PAAc-CaAc$_2$ hydrogels with different cross-linker densities. (A–B) Stress-strain curves for (A) as-prepared PAAc gels and (B) equilibrated PAAc-CaAc$_2$ gels at *r.t.* with different cross-linker densities at a deformation rate of 100 mm/min. The insets in (A) and (B) show a magnified view in the small-strain region. The monomer concentration in as-prepared state was 3 M; (C) The equilibrium volume ratio, $Q_V = V_{eq}/V_{as\text{-}pre}$, where V_{eq} and $V_{as\text{-}pre}$ are the volumes of the equilibrated PAAc-CaAc$_2$ and as-prepared PAAc hydrogels at *r.t.*, respectively. The equilibrated gels were prepared by immersing the as-prepared gels in a 250 mM calcium acetate (CaAc$_2$) solution.

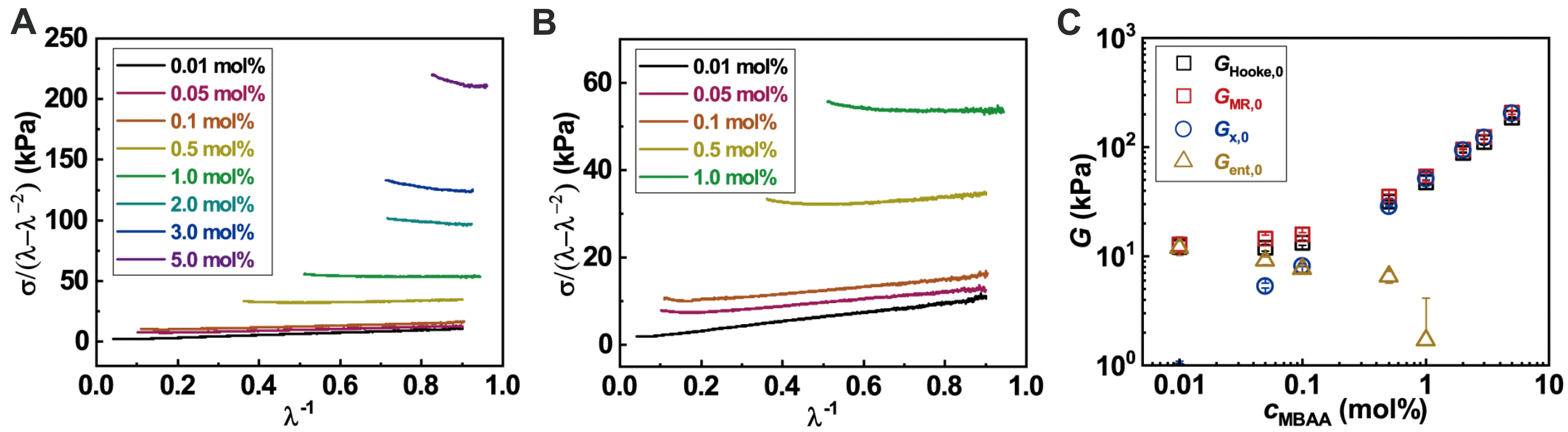

Fig. S4. Estimation of shear modulus from permanent cross-links. (A-B) Mooney-Rivlin plots of as-prepared PAAc gels from tensile data in Fig. S3A, with panel (B) providing a magnified view of the low-stress region; (C) Comparison of shear modulus obtained via different analytical methods. $G_{\mathrm{Hooke},0}$ represents the shear modulus estimated from the initial linear slope of stress-strain curves ($\varepsilon < 0.1$), assuming an incompressible network with $G_{\mathrm{Hooke},0} = E_{\mathrm{Hooke},0}/3$; $G_{\mathrm{MR},0}$ represents the shear modulus derived from Mooney-Rivlin plots ($G_{\mathrm{MR},0} = G_{\mathrm{x},0} + G_{\mathrm{ent},0}$), where $G_{\mathrm{x},0}$ and $G_{\mathrm{ent},0}$ denote contributions from permanent cross-linking and transient entanglements, respectively. The close agreement between $G_{\mathrm{MR},0}$ and $G_{\mathrm{Hooke},0}$ validates the application of the Mooney-Rivlin framework to these hydrogel systems.

Methodological Note: Extraction of $G_{\mathrm{x},0}$ and Scaling to G_{x}

The permanent and transient components of the modulus were decoupled using the Mooney–Rivlin relationship for the pre-hardening region: $\sigma/(\lambda-\lambda^{-2}) = 2C_1 + 2C_2\lambda^{-1}$. Through linear extrapolation, the intercept $2C_1$ is assigned to the permanent network shear modulus ($G_{\mathrm{x},0}$), while the slope $2C_2$ reflects transient contributions $G_{\mathrm{ent},0}$, primarily arising from constrained chain fluctuations and transient entanglements (*25–27*).

In the crowded PAAc-$CaAc_2$ hydrogels, the permanent network modulus (G_{x}) is considered to impose the dominant elastic energy penalty on phase separation. We estimated G_{x} by scaling $G_{\mathrm{x},0}$ to account for the volume contraction Q_{V} (= $V_{\mathrm{eq}}/V_{\mathrm{as\text{-}pre}}$) using the classical affine model: $G_{\mathrm{x}} = G_{\mathrm{x},0} \times Q_{\mathrm{V}}^{-1/3}$

Alternative scaling models that incorporate concentration-dependent chain fluctuations in unentangled networks ($G_{\mathrm{x}} = G_{\mathrm{x},0} \times Q_{\mathrm{V}}^{-7/12}$) yield only marginal differences and do not alter the fundamental conclusions of this work *(28)*. Consequently, the affine-scaled G_{x} was utilized throughout the manuscript to represent the permanent elastic constraint of the system.

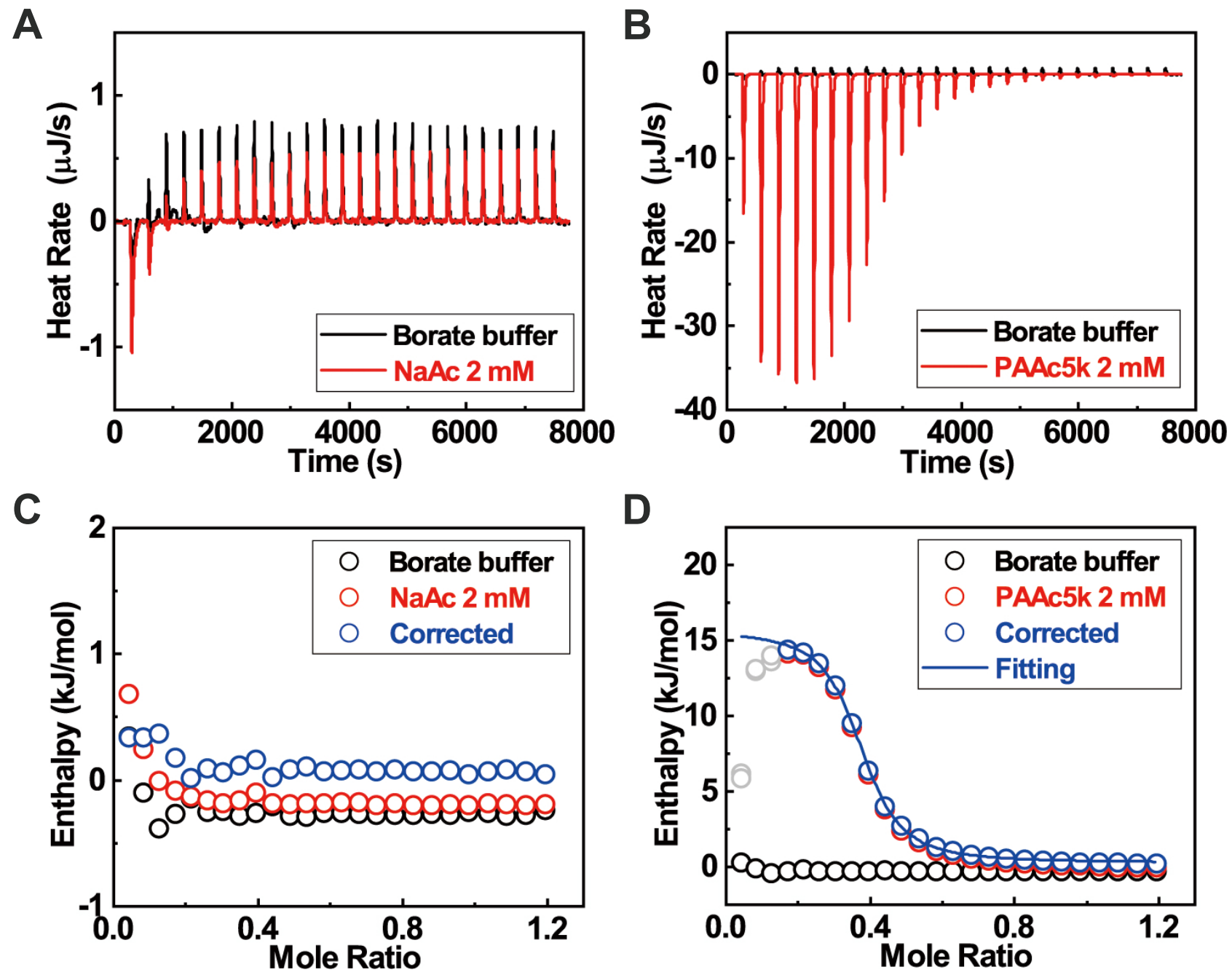

Fig. S5. ITC measurements of $CaCl_2$ titrated into various solutions at 25 °C. (A, C) Corrected heat rate and enthalpy per injection for $CaCl_2$ solution titrated into borate buffer and sodium acetate (NaAc) solutions, indicating the negligible complexation between Ca^{2+} and Ac^-; (B, D) Corrected heat rate and enthalpy per injection for $CaCl_2$ to buffer and polyacrylic acid (PAAc, M_w = 5000) solutions. Upward deflection indicates exothermic in (A) and (B). The blue hollow circles in (C) and (D) represent the data after subtraction of the dilution heat of $CaCl_2$ into buffer. All solutions were prepared using 5 mM borate buffer, with the pH maintained at 9.02 ± 0.01. The concentration of $CaCl_2$ was 8.0 mM and the concentrations of NaAc and PAAc repeat unit were 2.0 mM. The independent binding model (A + B ⇌ AB) was adopted for fitting the ITC data, shown as blue curve. The first three injections were excluded from the fitting and are shown as gray circles.

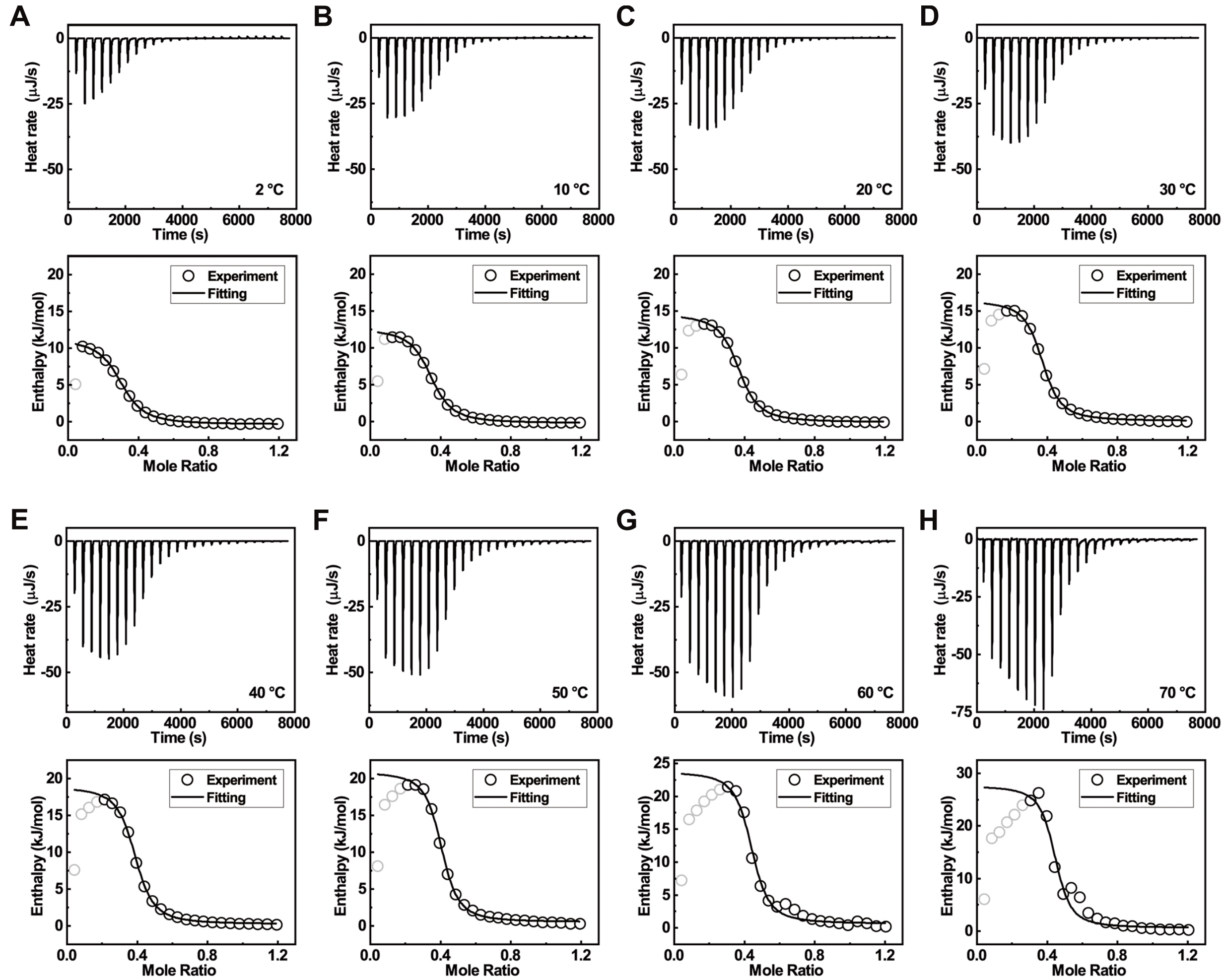

Fig. S6. ITC measurements of $CaCl_2$ binding to carboxylate sites on PAAc chains at various temperatures. (A) 2 °C; (B) 10 °C; (C) 20 °C; (D) 30 °C; (E) 40 °C; (F) 50 °C; (G) 60 °C; (H) 70 °C. Upward deflection indicates exothermic heat flow in each top panel. All solutions were prepared using 5 mM borate buffer, with the pH maintained at 9.02 ± 0.01 at 25 °C. The $CaCl_2$ and PAAc repeat-unit concentrations were 8.0 mM and 2.0 mM, respectively (M_w of PAAc= 5000). The heat of the final injection was used as the background and subtracted during fitting. The independent binding model (A + B ⇌ AB) was adopted for fitting the ITC data. The first several injections were excluded from the fitting and are shown as gray circles.

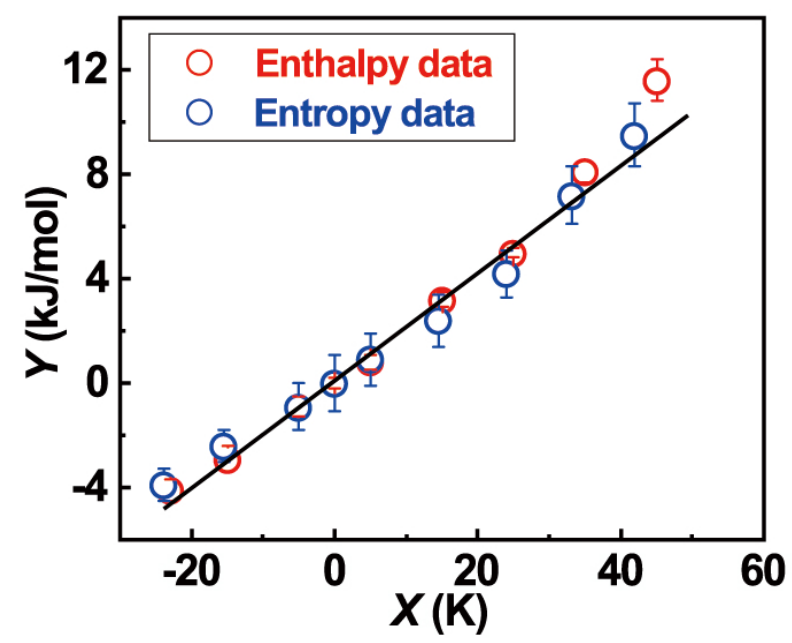

Fig. S7. Extraction of the heat-capacity change ΔC_p from the temperature dependence of PAAc-Ca^{2+} complexation, using T_0 = 25 °C as the reference temperature. Enthalpy data (red circles) are plotted as $Y = \Delta H - \Delta H_0$ versus $X = T - T_0$, and entropy data (blue circles) as $Y = T_0(\Delta S - \Delta S_0)$ versus $X = T_0 \ln (T/T_0)$, such that the slope yields ΔC_p. A unified fit to both datasets (black line) yielded $\Delta C_p = (0.20 \pm 0.01)$ kJ/(mol·K).

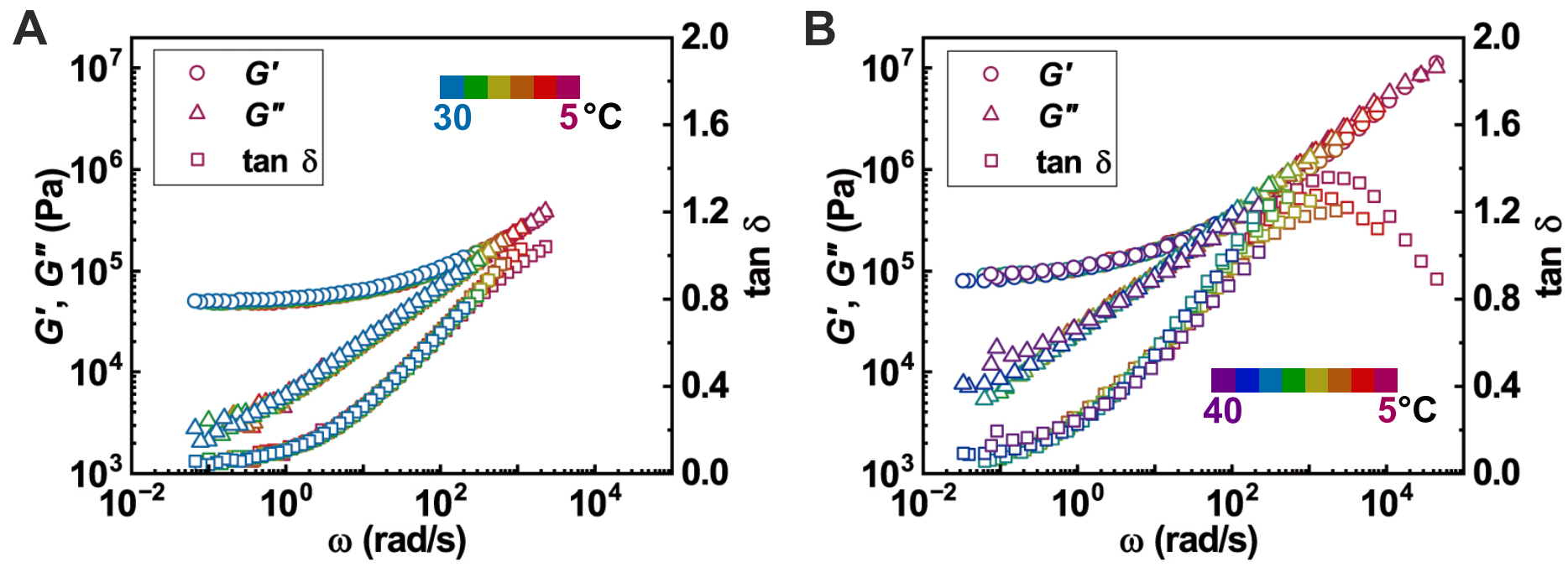

Fig. S8. Time-temperature superposition (TTS) rheological analysis of crowded hydrogels at varying water contents. (A) Sample with 46 wt% water content, initially equilibrated at T_{eq} = 30 °C; (B) Sample with 41 wt% water content, initially equilibrated at T_{eq} = 40 °C. Frequency sweep measurements were performed within a temperature range below the respective demixing temperature (T_{dem}) to maintain a homogeneous state. TTS master curves were constructed by superposing these frequency-sweep curves at a reference temperature of 25 °C. To satisfy the TTS requirement of constant composition, samples were submerged in paraffin oil during testing to prevent water evaporation. The cross-linking density of hydrogel is 1.0 mol%.

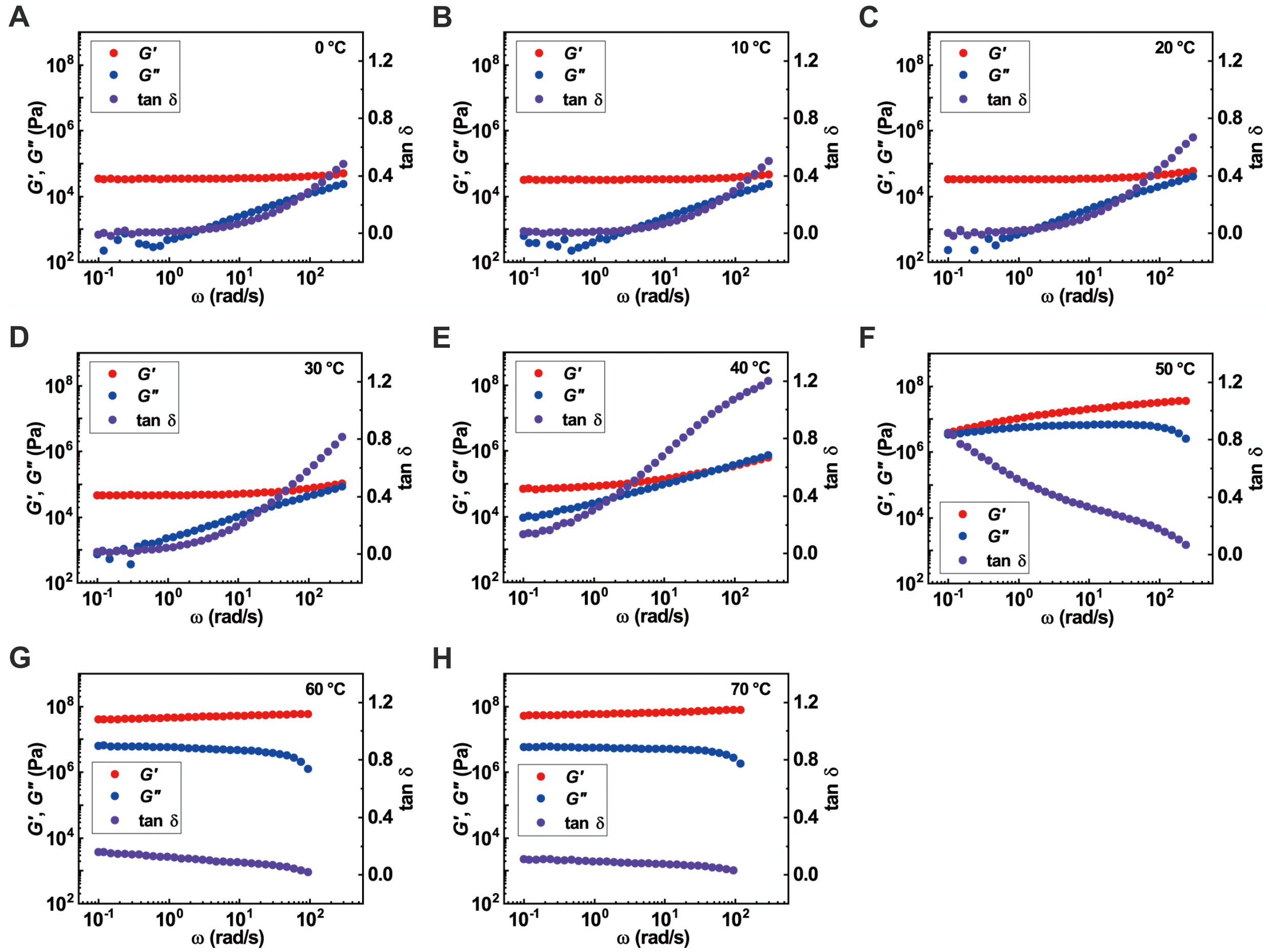

Fig. S9. Rheological properties of PAAc-CaAc$_2$ hydrogels with 1.0 mol% cross-linker conditioned at different temperatures. The hydrogels are first heated to different temperatures via a quasi-static heating pathway (0.1 °C/h), followed by frequency sweeps performed at their respective soaking temperatures. (A−H) G', G'', and tan δ for gels conditioned at (A) 0 °C; (B) 10 °C; (C) 20 °C; (D) 30 °C; (E) 40 °C; (F) 50 °C; (G) 60 °C; (H) 70 °C. Frequency sweep tests were performed over a frequency range of 0.1 to 300 rad/s at a strain of 0.1% for gels. For the gels equilibrated below 50 °C, their G' exhibited a plateau at low frequency.

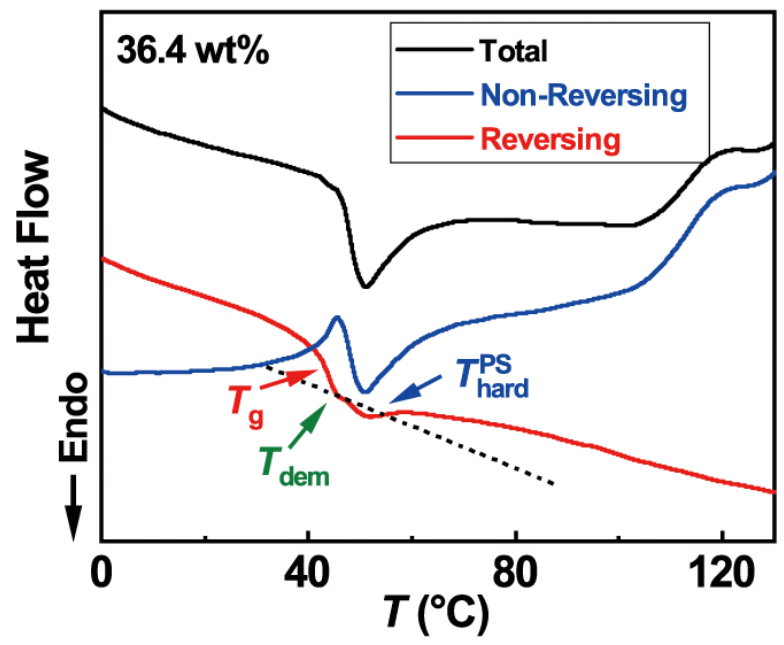

Fig. S10. Representative MDSC results of PAAc-$CaAc_2$ hydrogels with 1.0 mol% cross-linker containing 36.4 wt% water. The red trace represents reversing heat flow, which reflects the phase transition affecting the heat capacity. On the red trace, the glass transition points for samples before demixing transition are indicated by red arrows. The black dotted line after glass transition represents the extrapolated baseline heat capacity. The deviation from the baseline guided by green arrow indicates the beginning of phase separation. The drop in heat capacity below the baseline guided by blue arrow indicates the beginning of hardening transition. MDSC traces were recorded at an average heating rate of 3 °C /min.

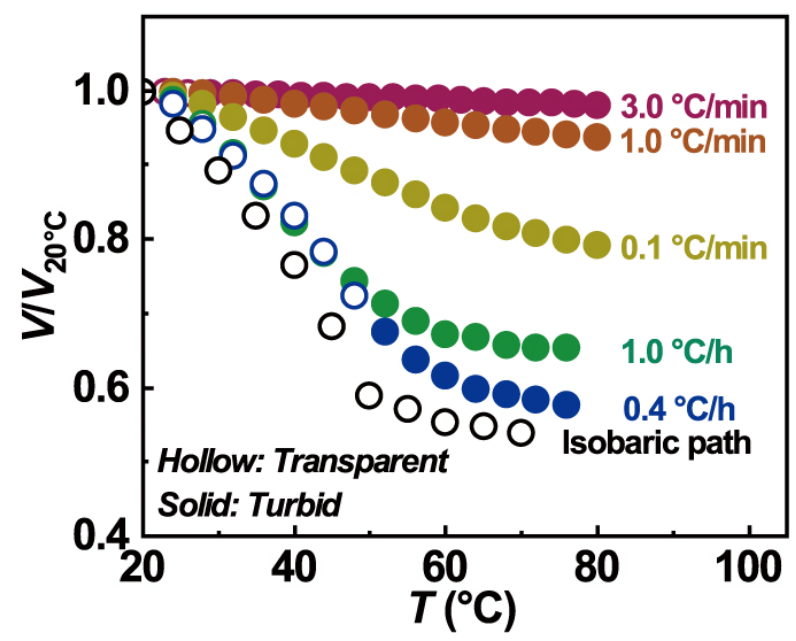

Fig. S11. Volume changes of PAAc-CaAc$_2$ hydrogels with 1.0 mol% cross-linker at different heating rates. The hydrogels are pre-equilibrated at 20 °C before heating. Hollow and solid circles represent the transparent and turbid states, respectively. The black circles represent the steady-state volume under quasi-isobaric conditions at a rather slow rate of 0.1 °C/h. During heating at rates above 1.0 °C/min, the normalized volume, $V/V_{20\,°C}$, remains nearly constant up to 80 °C, indicating a quasi-isochoric condition. The equilibrium thickness of gels at 20 °C was 1.1 mm.

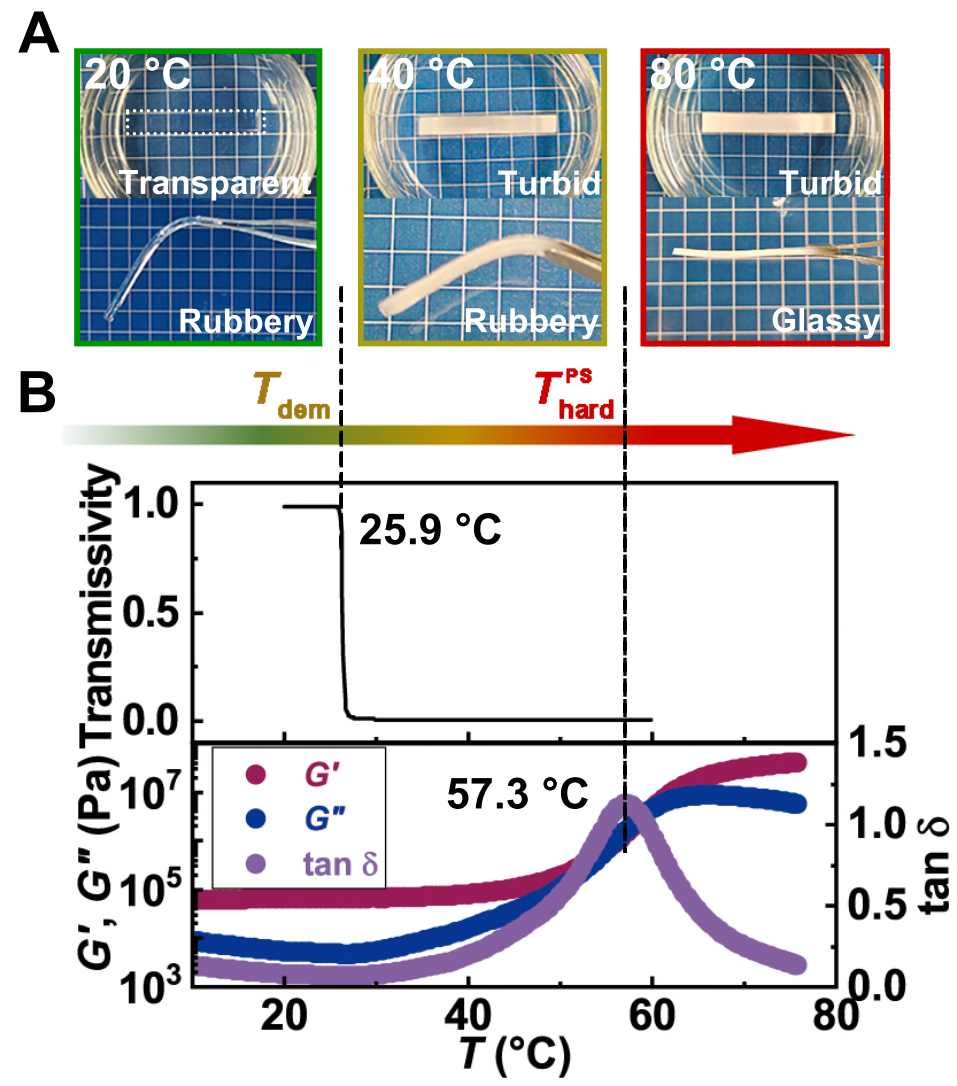

Fig. S12. Kinetic-driven thermal demixing and hardening transitions of PAAc-$CaAc_2$ hydrogels. (A) Representative photo of gels in three states during heating from 20 °C at a rate of 2 °C/min; (B) Heating curves for UV-vis and rheology of gels. Heating ramps of UV-vis spectrum and rheology were 2 °C/min and 3 °C/min, respectively. Demixing temperature (T_{dem}) and hardening temperature ($T^{\mathrm{PS}}_{\mathrm{hard}}$) were determined from the abrupt decrease in transmittance (95% transmittance) and the peak of tan δ, respectively. The PAAc-$CaAc_2$ gels (1.0 mol% cross-linker, ≈ 1.1 mm thick) were pre-equilibrated at 20 °C.

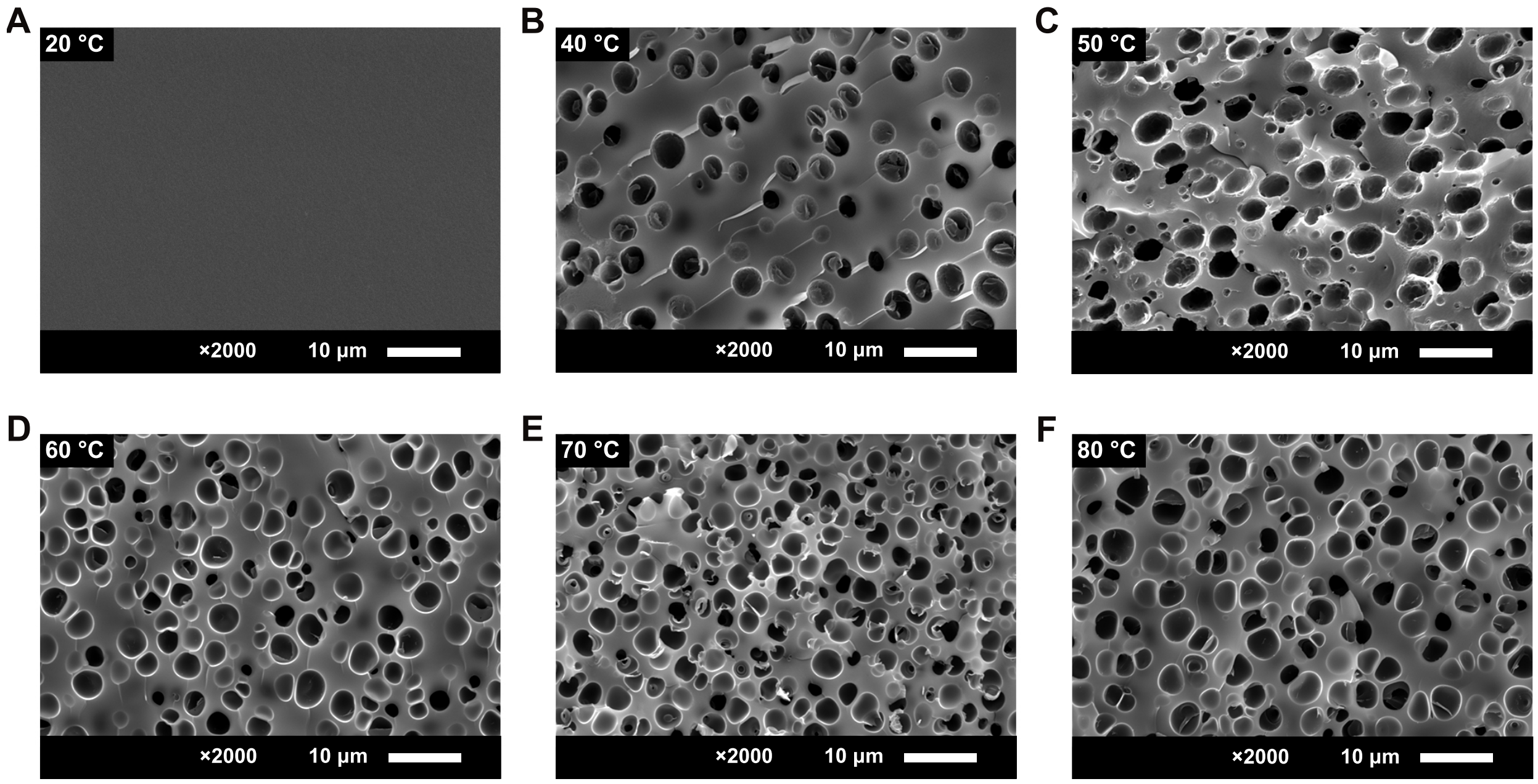

Fig. S13. SEM images of gels heated from 20 °C to different temperatures under isochoric conditions. (A) 20 °C; (B) 40 °C; (C) 50 °C; (D) 60 °C; (E) 70 °C; (F) 80 °C. The magnification is ×2000 (scale bar: 10 μm). Hydrogels are pre-equilibrated at 20 °C, then heated to different temperatures at a rate of 2 °C/min. After freeze-drying, the cross-sectional structures were observed by SEM. The cross-linking density of hydrogel is 1.0 mol%. The equilibrium thickness of gels at 20 °C was around 1.1 mm.

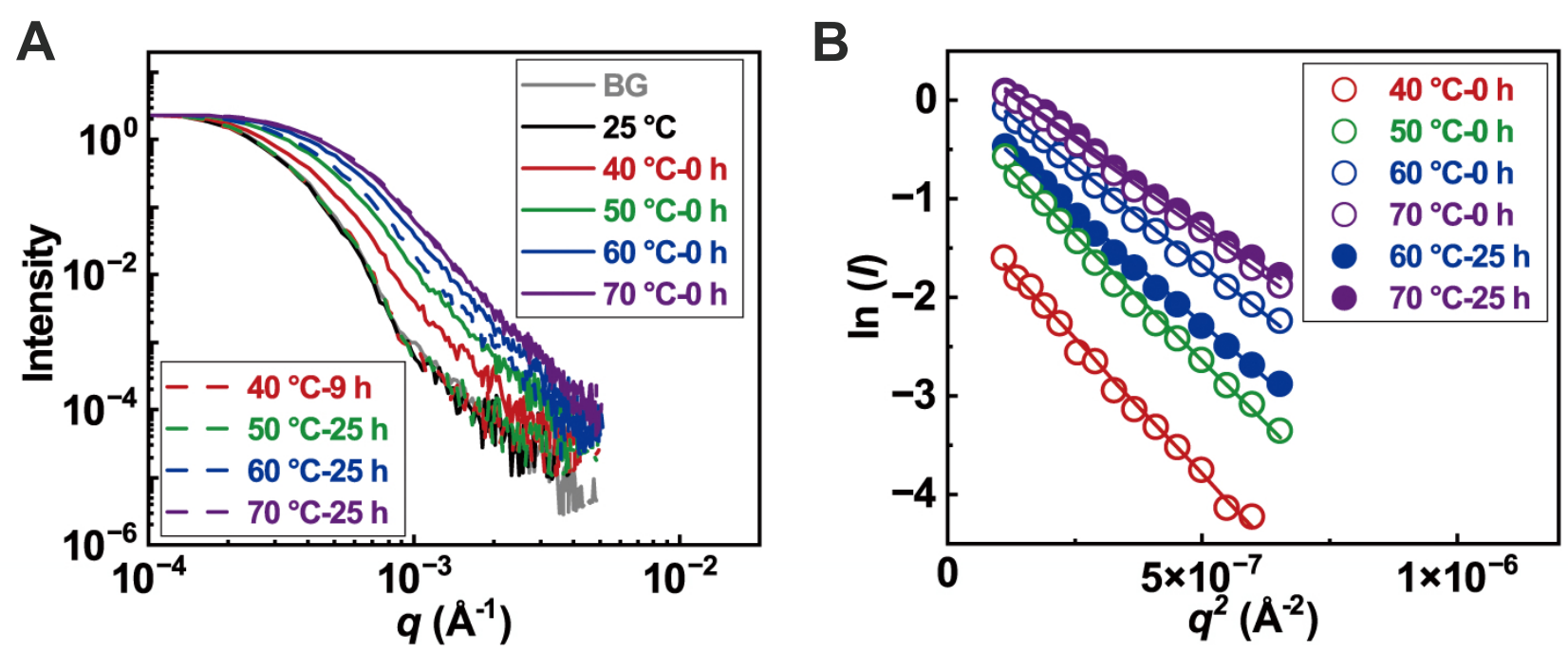

Fig. S14. USANS analysis of PAAc-$CaAc_2$ hydrogels at different temperatures. (A) The USANS profiles. The isothermal temperatures are 40 °C (red curves), 50 °C (green curves), 60 °C (blue curves) and 70 °C (purple curves). The solid curves represent the profiles immediately after the *T*-jump to the destination temperature, while the dashed curves represent the profiles after prolonged isothermal annealing. The gray solid curve represents the background (BG) of the empty cell. The black solid curve represents the profiles of hydrogel at *r.t.*, where no phase separation occurs. Hydrogel had a chemical cross-linker density of 1.0 mol% and an equilibrium thickness of 0.8 mm at 20 °C. The change in intensity reflects the density contrast between the two phases generated by phase separation, with higher intensity indicating a larger density difference. At 40 °C and 50 °C, the gel reaches equilibrium after 9 h and 25 h. At 60 °C and 70 °C, after 25 h of annealing, the intensity shows little variation, indicating that phase separation persists and does not vanish; (B) Guinier plots. According to the Guinier equation S.1, the data were fitted over the range $q = (2.5 \sim 8.0) \times 10^{-4}$ Å^{-1}. The radius of gyration, R_g is calculated from the slope. Hollow symbols represent the profiles immediately after *T*-jumping to the destination temperature, while the solid symbols represent the profiles after prolonged isothermal annealing. The radius of the scattering objects is obtained from the equation S.2.

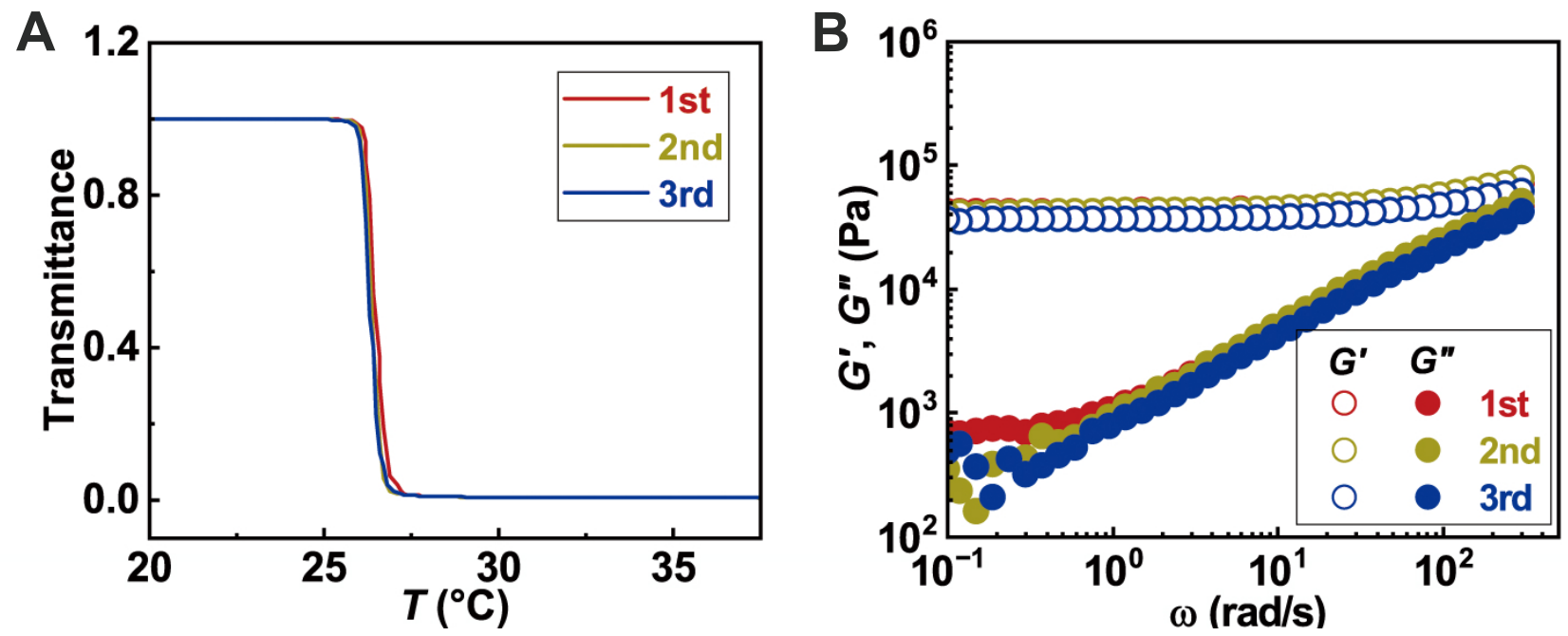

Fig. S15. Reversibility of hydrogel properties during repeated heating cycles with prescribed annealing time. (A) Three repeated transmittance measurements upon heating at a rate of 2 °C/min; (B) Three frequency sweeps performed at 20 °C with an applied strain of 0.1%.

The gel with 1.0 mol% cross-linker was soaked in a 20 °C solution until equilibrium. The red curve represents the first heating process. After that, the gel was re-soaked in the 20 °C solution for 24 h and tested again with the same heating process (yellow curve). After an additional 24 h of annealing at 20 °C, the measurement was repeated on the third day (blue curve). The consistent T_{dem} and mechanical properties indicate that there is no irreversible damage to the internal network during phase separation. The equilibrium thickness of the gel at 20 °C was around 1.1 mm.

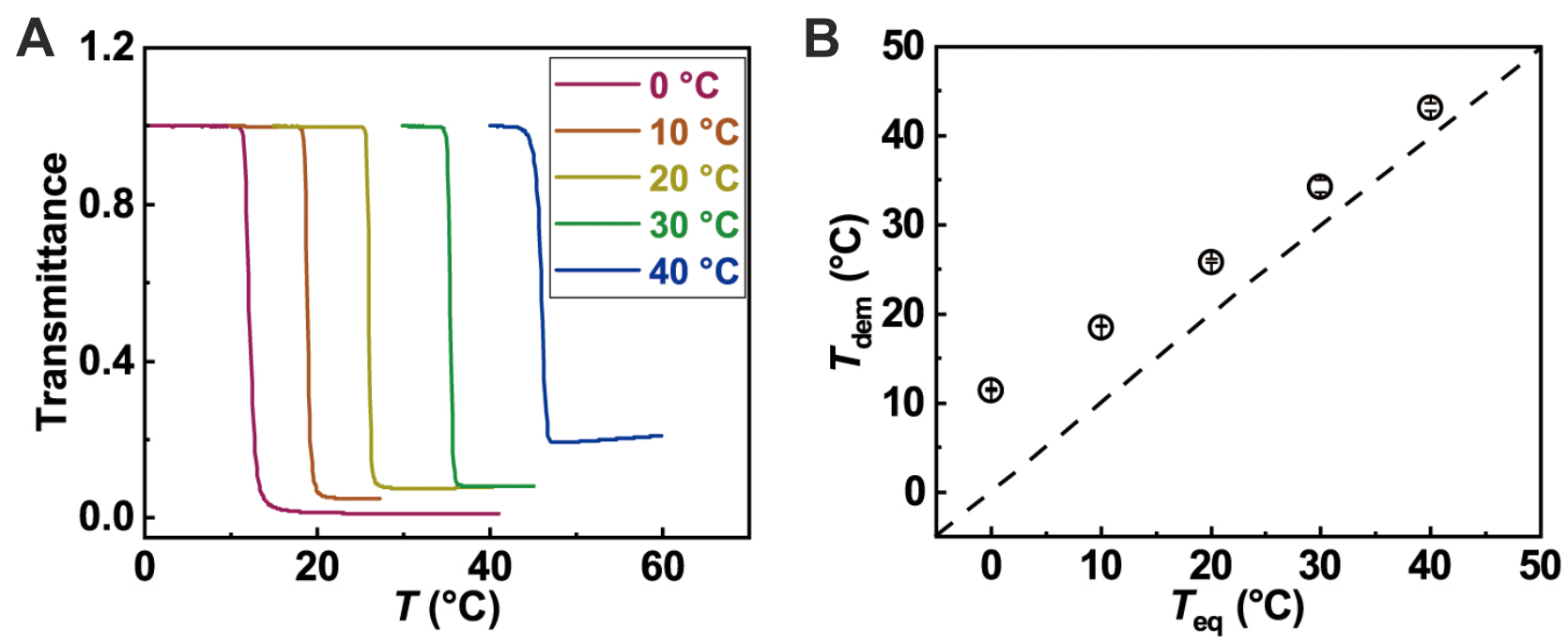

Fig. S16. Phase separation upon heating for PAAc-$CaAc_2$ hydrogels with 1.0 mol% cross-linker. (A) Change in transmittance upon heating for equilibrium gels pre-equilibrated in $CaAc_2$ solutions of different temperatures T_{eq} (red: 0 °C; orange: 10 °C; yellow: 20 °C; green: 30 °C; blue: 40 °C); (B) T_{dem} as a function of equilibrium temperature T_{eq}. The dashed line is for the case when T_{dem} equals T_{eq}. The heating rate was 2 °C/min. The thickness of the gels equilibrated at 20 °C was around 1.1 mm.

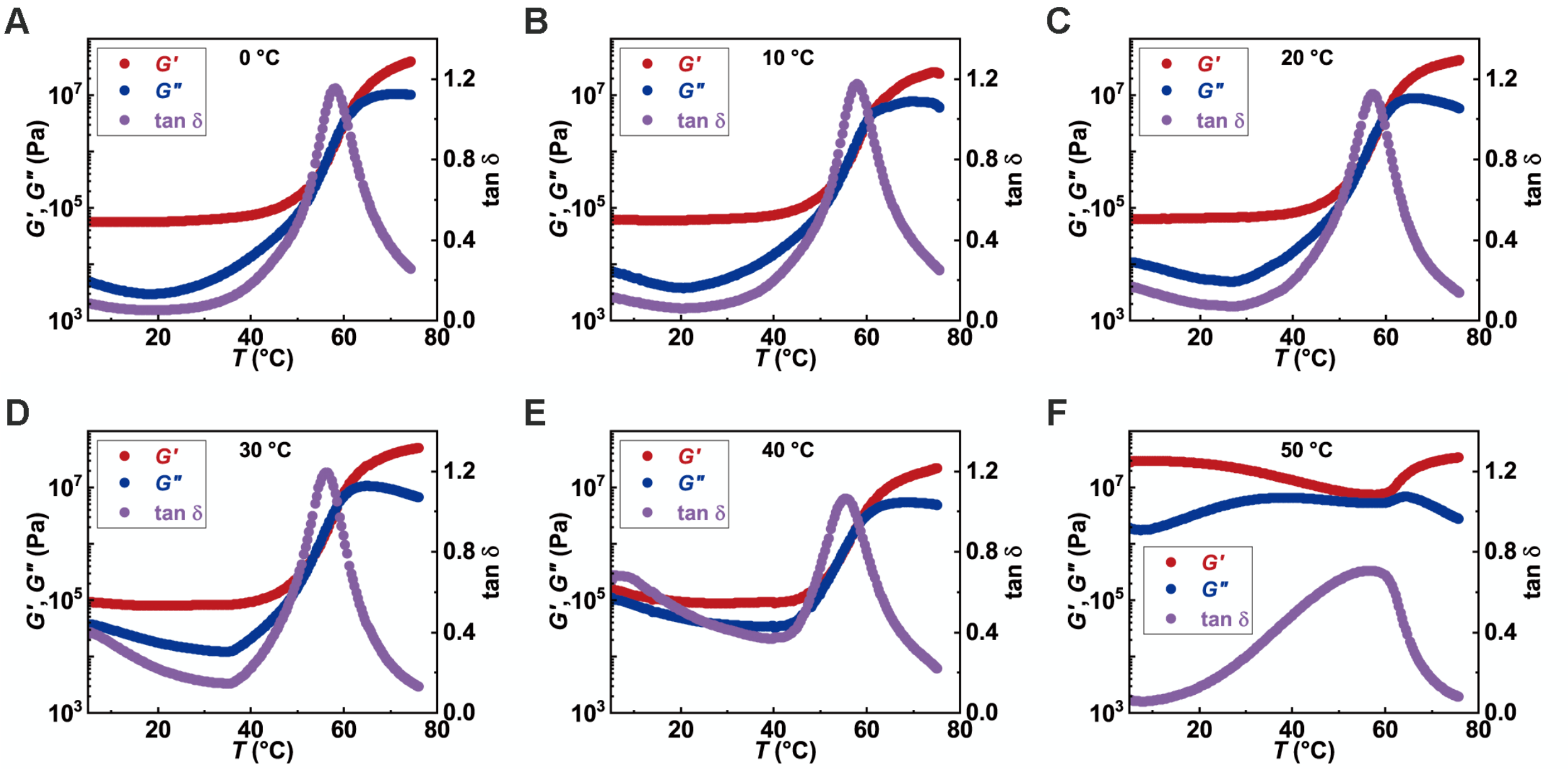

G

T_{eq} (°C)	T_{hard}^{PS} (°C)
0	57.8
10	57.9
20	57.4
30	56.9
40	55.4
50	58.0

Fig. S17. Rheological behaviors of PAAc-$CaAc_2$ hydrogels in response to a fast temperature sweep. The gels with 1.0 mol% cross-linker density were equilibrated at different temperatures T_{eq} below T_{hard}^{PS}. The samples were then rapidly cooled to 5 °C and heated to 80 °C at a rate of 3 °C/min. The results correspond to equilibrium temperatures T_{eq} at (A) 0 °C, (B) 10 °C, (C) 20 °C, (D) 30 °C, (E) 40 °C, (F) 50 °C; (G) T_{hard}^{PS} obtained from the peak of tan δ of (A–F). Temperature sweep was performed at 10 rad/s. The thickness of the gels equilibrated at 20 °C was around 1.1 mm.

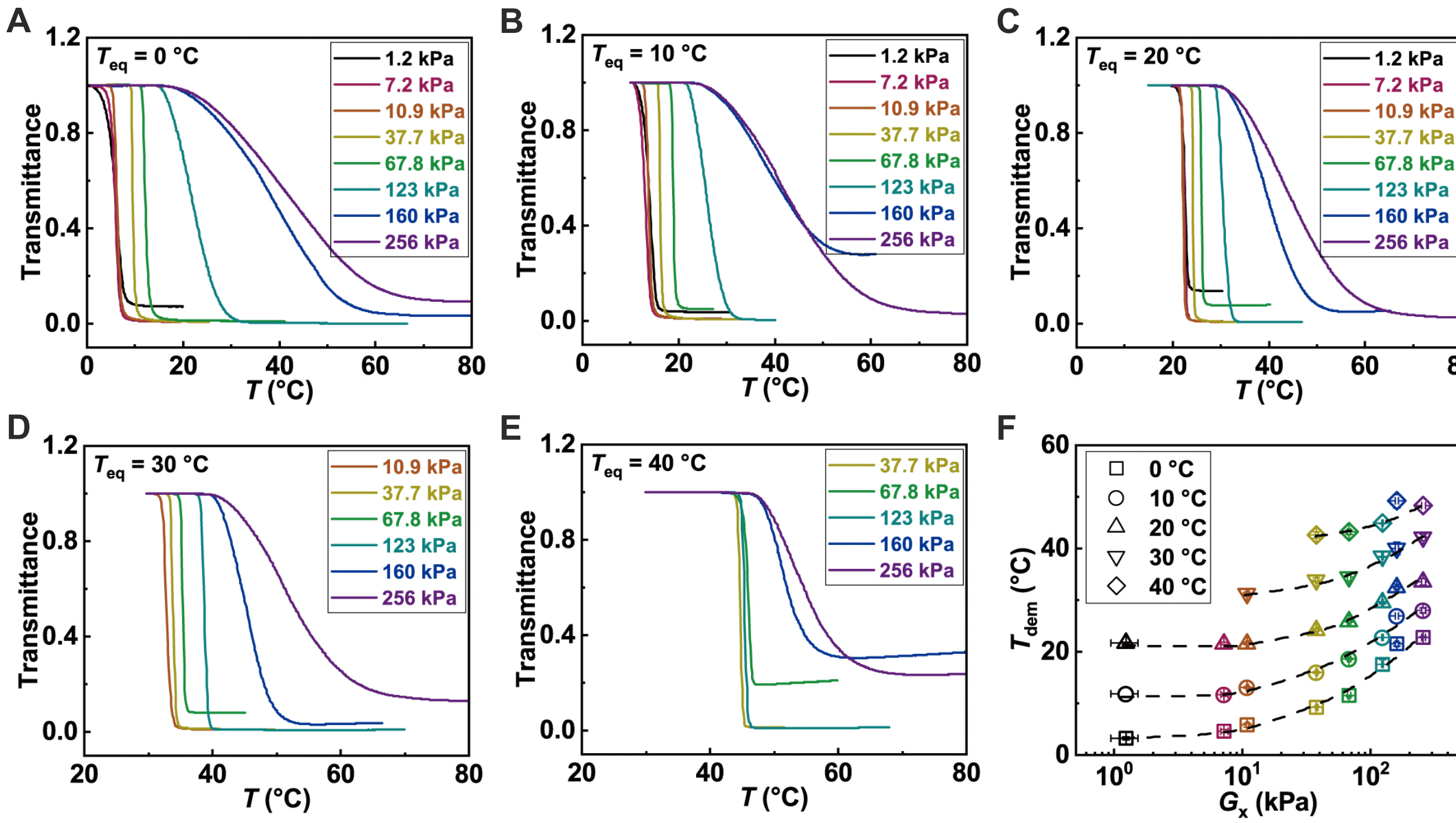

Fig. S18. Transmittance change and demixing temperature T_{dem} upon fast heating for PAAc-$CaAc_2$ hydrogels with different G_x. (A–E) Transmittance change upon heating for gels equilibrated in $CaAc_2$ solutions at (A) 0 °C, (B) 10 °C, (C) 20 °C, (D) 30 °C and (E) 40 °C; (F) T_{dem} as a function of the permanent network shear modulus, G_x, for gels equilibrated at different temperatures T_{eq} as indicated in the figure. The heating rate was 2 °C/min. The thickness of the gels equilibrated at 20 °C was around 0.8 mm.

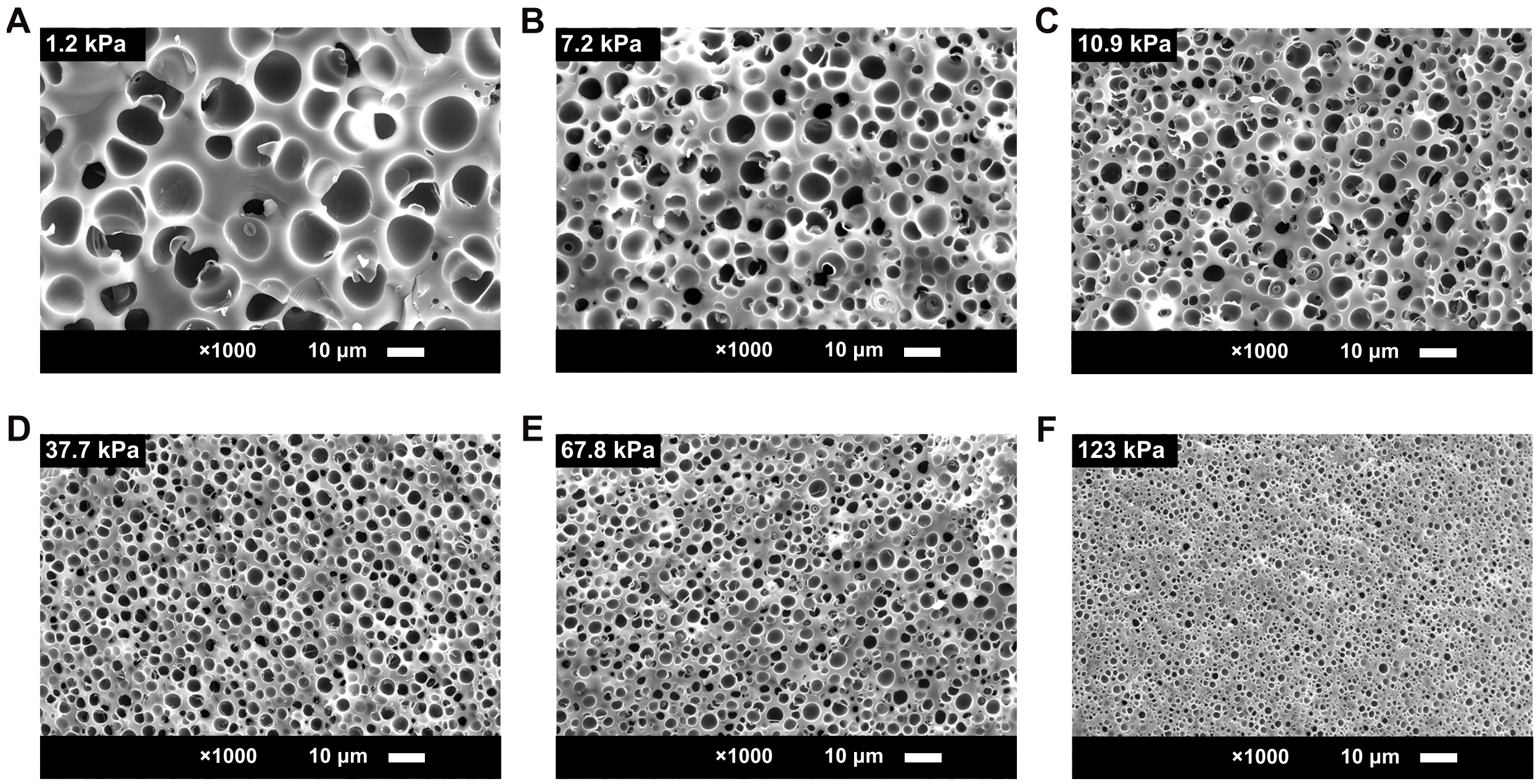

Fig. S19. SEM images showing micropores in PAAc-CaAc$_2$ hydrogels with different G_x controlled by cross-linker densities after fast heating. The cross-linker density is (A) 0.01 mol%; (B) 0.05 mol%; (C) 0.1 mol%; (D) 0.5 mol%; (E) 1.0 mol%; (F) 2.0 mol% relative to the monomer concentration. Hydrogels are pre-annealed at 20 °C until equilibrium, then heated to 80 °C at a rate of 2 °C/min. After freeze-drying, the cross-sectional structures were observed by SEM. The magnification is ×1000 (scale bar: 10 µm). The equilibrium thickness of gels at 20 °C was around 0.8 mm. Labels indicate G_x at room temperature.

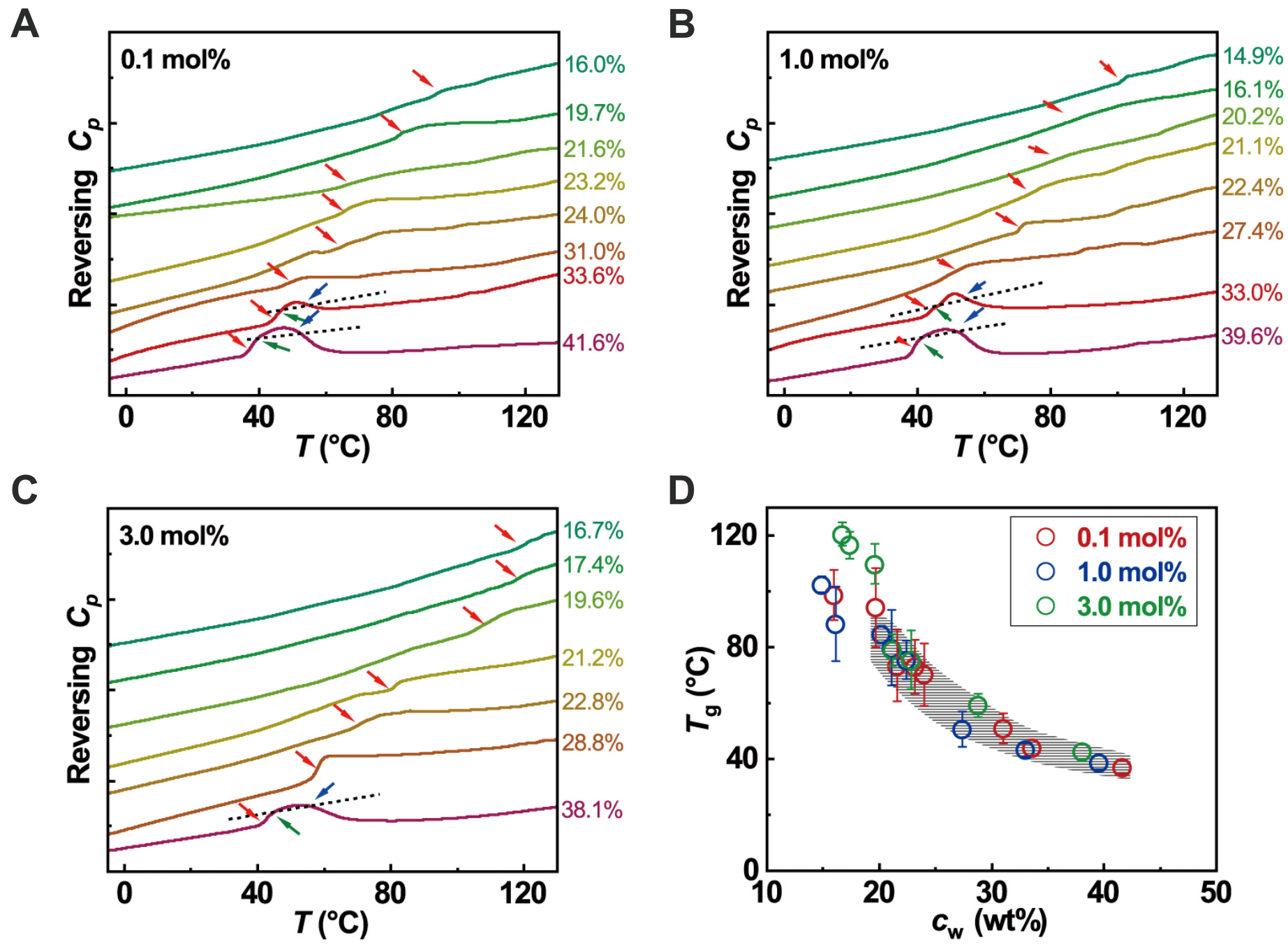

Fig. S20. Effect of chemical cross-linker density on the glass transition temperature of PAAc-$CaAc_2$ hydrogels with varied water contents. (A–C) Reversing heat capacity curves obtained from MDSC measurements for hydrogels with cross-linker densities of 0.1 mol%, 1.0 mol% and 3.0 mol%, respectively, with different water contents, c_w. The numbers on the right denote the c_w values corresponding to each curve. The red arrows indicate the glass transition temperatures. (D) Glass transition temperature T_g as a function of c_w, extracted from panels (A–C). The shaded region highlights that the three hydrogels exhibit nearly identical T_g profiles when the water content exceeds 20 wt% (the primary regime of interest in this study), demonstrating that T_g is effectively independent of cross-linker density in this range. The permanent-network shear moduli (G_x) for the 0.1 mol%, 1.0 mol%, and 3.0 mol% samples are 10.9 kPa, 67.8 kPa, and 160 kPa, respectively. The equilibrium thickness of the samples at 20 °C was around 0.8 mm.

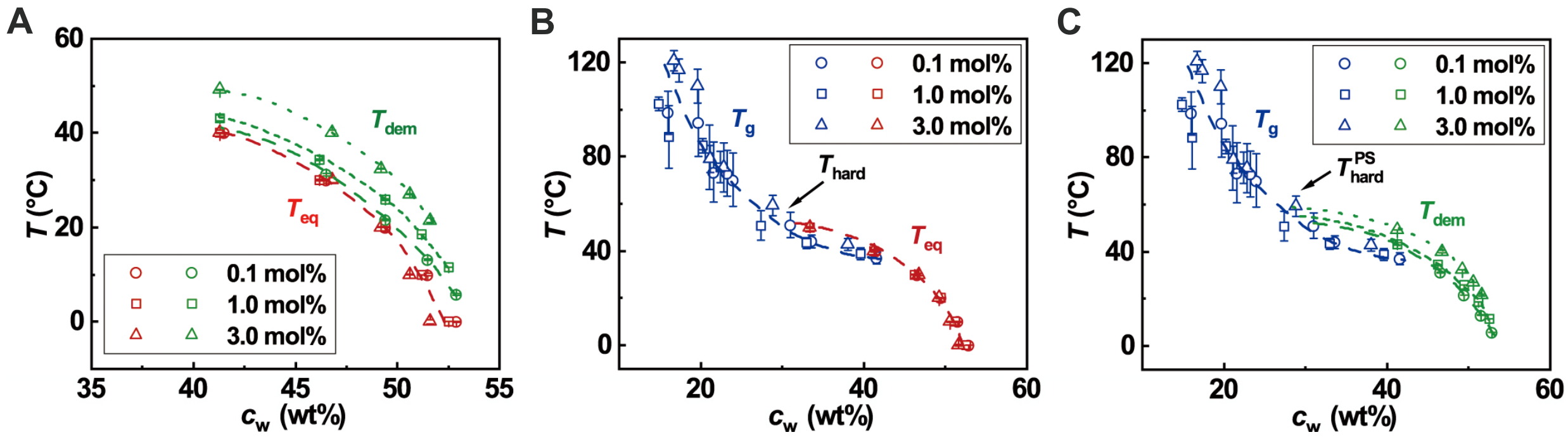

Fig. S21. Phase diagrams of PAAc-$CaAc_2$ hydrogels with different cross-linker densities. (A) Thermodynamic phase diagram in the temperature-water content (T-c_w) space. Red symbols represent the equilibrium water content $c_{w,eq}$ as a function of T_{eq}, while green symbols denote the corresponding demixing temperatures T_{dem} obtained at the same water contents. Circles, squares, triangles correspond to hydrogels with cross-linker densities of 0.1 mol%, 1.0 mol%, and 3.0 mol%, respectively. Dashed curves are drawn as visual guides. The equilibrium curves of hydrogels with different cross-linker densities nearly overlap and are therefore represented by a single dashed curve; (B) Hardening temperature T_{hard} defined by the intersection between the glass transition T_g-c_w curve and the thermodynamic equilibrium T_{eq}-$c_{w,eq}$ curve in the temperature-water content space; (C) Phase-separation hardening temperature T_{hard}^{PS} defined by the intersection between the glass transition T_g-c_w curve and the demixing curve T_{dem}-c_w. In panels (B–C), the blue dashed curve represents the glass transition curve shared by the three hydrogels. The red dashed curve represents the overlapping equilibrium curve, indicating that the resulting T_{hard} values are nearly identical for the three hydrogels. The green dashed, short-dashed, and dotted curves represent the demixing curves for hydrogels with cross-linker densities of 0.1 mol%, 1.0 mol%, and 3.0 mol%, respectively, showing that the phase-separated hardening temperature T_{hard}^{PS} increases with cross-linker density. The permanent-network shear moduli (G_x) for the 0.1 mol%, 1.0 mol% and 3.0 mol% samples are 10.9 kPa, 67.8 kPa, and 160 kPa, respectively. The equilibrium thickness of the gels at 20 °C was around 0.8 mm.

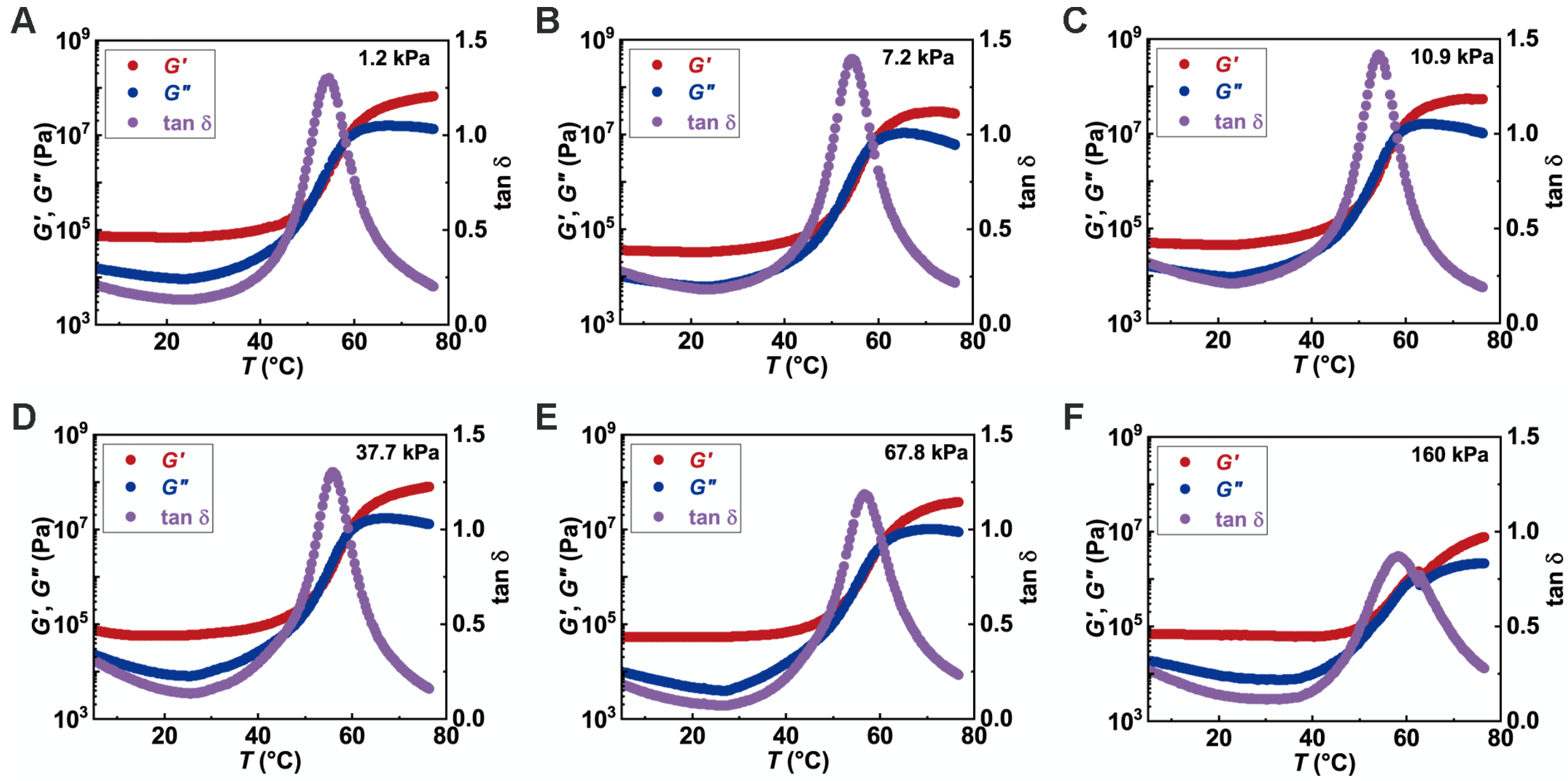

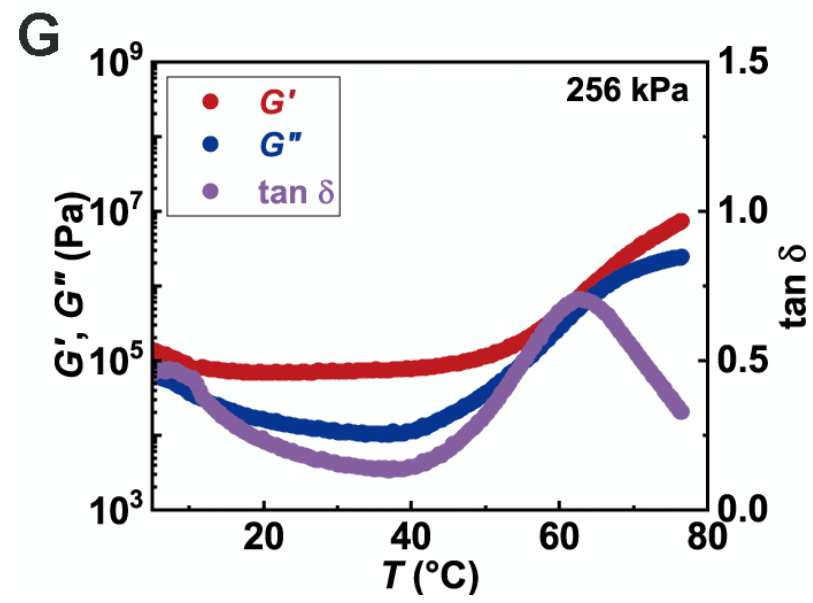

H

c_{MBAA} (mol%)	G_x (kPa)	T_{hard}^{PS} (°C)
0.01	1.2	54.5
0.05	7.2	54.3
0.1	10.9	54.3
0.5	37.7	56.1
1.0	67.8	56.8
3.0	160	58.4
5.0	256	62.9

Fig. S22. Rheological behaviors of PAAc-CaAc$_2$ hydrogels in response to fast heating. The heating was performed for gels with different shear moduli G_x equilibrated at 20 °C. The temperature range is from 5 °C to 80 °C at a rate of 3 °C/min; Temperature sweep tests were performed for hydrogels with G_x = (A) 1.2 kPa, (B) 7.2 kPa, (C) 10.9 kPa, (D) 37.7 kPa, (E) 67.8 kPa, (F) 160 kPa, (G) 256 kPa. (H) T_{hard}^{PS} obtained from the tan δ peaks in panels (A–G). The equilibrium thickness of the gels at 20 °C was around 0.8 mm.

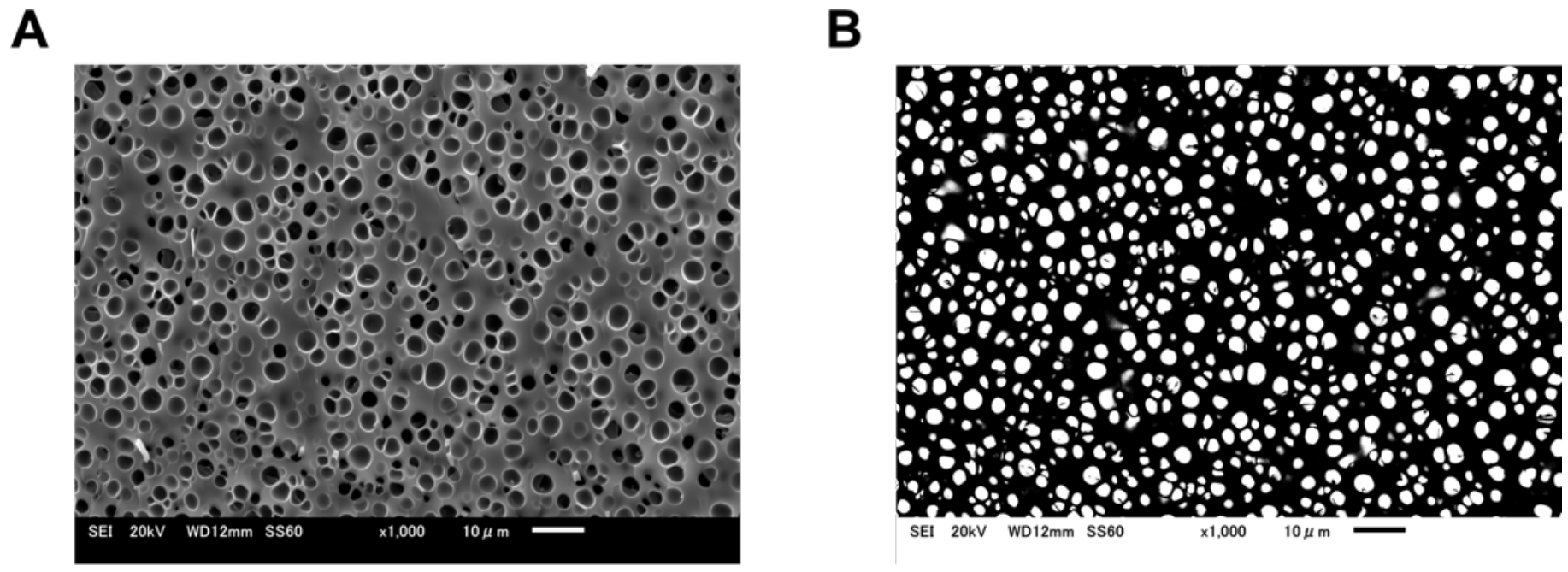

Fig. S23. The original SEM image (A) and the binarized image (B) for hydrogels heating to 60 °C. The SEM image corresponds to the same region as shown in Fig. S13D, but at a different magnification.

Table S1. Experimental parameters for PAAc gels in salt solutions.

Salt	*c*/ mM	pH (initial)	pH (equilibrium)	Q_V*
**NaAc**	750	7.968	5.436	3.82
**$CaAc_2$**	250	7.289	5.132	0.45

*Q_V is the volume ratio of gels, defined as $Q_V = V_{eq}/V_{as\text{-}pre}$. V_{eq} and $V_{as\text{-}pre}$ are the volumes in the equilibrium and as-prepared states at *r.t.*, respectively. We calculated the volume ratio from the diameter change of the disc-shaped hydrogel, *i.e.*, $Q_V = V_{eq}/V_{as\text{-}pre} = (d_{eq}/d_{as\text{-}pre})^3$.

Table S2. Shear modulus from permanent cross-links for as-prepared PAAc hydrogels with different chemical cross-linker densities at *r.t.* ($G_{x,0}$), and the corresponding values for the equilibrium PAAc-$CaAc_2$ hydrogels (G_x).

c_{MBAA}/mol%	$G_{x,0}$/kPa	Q_V	G_x^*/kPa
**0.01**	0.9 ± 0.2	0.369	1.2 ± 0.3
**0.05**	5.4 ± 0.3	0.422	7.2 ± 0.5
**0.1**	8.3 ± 0.2	0.437	10.9 ± 0.2
**0.5**	28.8 ± 0.6	0.446	37.7 ± 0.7
**1.0**	51.8 ± 1.6	0.446	67.8 ± 2.1
**2.0**	95.2 ± 1.2	0.464	123 ± 2
**3.0**	123 ± 4	0.463	160 ± 5
**5.0**	206 ± 7	0.522	256 ± 9

$^*G_x = G_{x,0} \times Q_V^{-1/3}$.

Table S3. Thermodynamic parameters for the binding of Ca^{2+} and carboxylate groups on PAAc polymer chains determined by ITC.

***T*/ °C**	***K*$_a$/ 10^4 M^{-1}**	***n******	**Δ*H*/ kJ/mol**	**Δ*S*/ J/(mol·K)**	**Δ*G*/ kJ/mol****
**2**	4.02 ± 0.75	0.296	11.2 ± 0.3	129 ± 0.3	-24.2 ± 0.4
**10**	5.01 ± 0.52	0.337	12.4 ± 0.4	134 ± 0.3	-25.5 ± 0.2
**20**	5.24 ± 0.18	0.353	14.4 ± 0.3	139 ± 1.2	-26.5 ± 0.1
**25**	5.34 ± 1.17	0.359	15.3 ± 0.1	142 ± 1.8	-26.9 ± 0.6
**30**	5.96 ± 0.66	0.361	16.1 ± 0.2	145 ± 1.5	-27.7 ± 0.3
**40**	6.09 ± 0.84	0.371	18.5 ± 0.2	150 ± 1.6	-28.7 ± 0.4
**50**	7.63 ± 1.20	0.386	20.4 ± 0.1	156 ± 1.2	-30.2 ± 0.4
**60**	10.0 ± 0.8	0.420	23.4 ± 0.3	166 ± 2.0	-31.9 ± 0.2
**70**	12.0 ± 4.8	0.444	26.9 ± 0.7	174 ± 2.1	-32.9 ± 1.1

*n is the binding number of Ca^{2+} per repeat unit.

**$\Delta G = \Delta H - T\Delta S$.

Table S4. Equilibrium volume ratios (Q_V) and water content ($c_{w,eq}$) of PAAc-$CaAc_2$ gels with 1.0 mol% cross-linker at different temperatures (T_{eq})*.

T_{eq}/ °C	Q_V**	$c_{w,eq}$***/ wt%
**0**	0.522 ± 0.009	53.0 ± 1.3
**10**	0.500 ± 0.009	51.6 ± 1.4
**20**	0.468 ± 0.008	49.2 ± 0.3
**30**	0.420 ± 0.008	46.2 ± 0.4
**40**	0.366 ± 0.009	41.2 ± 0.2
**50**	0.274 ± 0.007	33.6 ± 1.1

*The hydrogel reached its final equilibrium volume after long-time annealing at T_{eq}.

**Q_V is defined to the as-prepared state of PAAc hydrogels.

***Water content was determined from the weight of equilibrated PAAc-$CaAc_2$ hydrogels at different T_{eq} before and after freeze-drying. The salt content was negligible.

Table S5. Size analysis of spherical polymer-dilute domains*.

***T*/°C**	***R_g* from USANS** /μm**	***R_{SANS}***** /μm**	***R_{SEM}* /μm**
**40**	0.407	0.525	1.54 ± 0.22
**50**	0.392	0.506	1.63 ± 0.17
**60**	0.349	0.450	1.59 ± 0.15
**70**	0.332	0.429	1.40 ± 0.15
**80**	/	/	1.66 ± 0.23
**60********	0.369	0.477	/
**70********	0.327	0.422	/

*The cross-linker density of the PAAc-$CaAc_2$ hydrogel was 1.0 mol%

**The USANS measurements were performed on different samples subjected to *T*-jump heating from 20 °C to each target temperature. The USANS chambers required more than 10 min to reach the target temperature, allowing the domains to fully develop. However, above 50 °C, the polymer dense matrix vitrified before the domains could grow to their maximum size, resulting in smaller values.

***For spherical domains, $R_{\mathrm{SANS}} = \sqrt{5/3}R_{\mathrm{g}}$

****The size after 25 h annealing at 60 or 70 °C.

Table S6. Comparison of pore/domain sizes and the characteristic mesh size of the network estimated from the modulus ($G_{x,0}$) of as-prepared PAAc hydrogels.

c_{MBAA} /mol%	$G_{x,0}$ /kPa	N^*	L_c^{**} (nm)	R_{SANS} (μm)	R_{SEM} (μm)
**0.01**	0.9	4258	1064	/	5.05
**0.05**	5.4	689	172	/	2.75
**0.1**	8.3	450	112	/	2.37
**0.5**	28.8	129	32	/	2.74
**1.0**	51.8	72	18	~0.5	1.68
**2.0**	95.2	39	10	/	0.92

*N represents the number of repeat units per strand, calculated by $G_{x,0} = \frac{c_M \times N_A}{2N} k_B T$, where c_M is the monomer concentration (3 M) before polymerization, $\frac{c_M \times N_A}{N}$ is the strand density.

**L_c is the contour length of polymer strands in the network, calculated by $L_c = Nl$, in which l is the length per repeat unit, roughly estimated as 0.25 nm.
